# A chromosome-level draft genome of the grain aphid *Sitobion miscanthi*

**DOI:** 10.1093/gigascience/giz101

**Published:** 2019-08-20

**Authors:** Xin Jiang, Qian Zhang, Yaoguo Qin, Hang Yin, Siyu Zhang, Qian Li, Yong Zhang, Jia Fan, Julian Chen

**Affiliations:** State Key Laboratory for Biology of Plant Diseases and Insect Pests, Institute of Plant Protection, Chinese Academy of Agricultural Sciences, Beijing 100193, People's Republic of China

**Keywords:** aphid, *Sitobion miscanthi*, *Sitobion avenae*, annotation, genome, long-read sequencing, Hi-C assembly

## Abstract

**Background:**

*Sitobion miscanthi* is an ideal model for studying host plant specificity, parthenogenesis-based phenotypic plasticity, and interactions between insects and other species of various trophic levels, such as viruses, bacteria, plants, and natural enemies. However, the genome information for this species has not yet to be sequenced and published. Here, we analyzed the entire genome of a parthenogenetic female aphid colony using Pacific Biosciences long-read sequencing and Hi-C data to generate chromosome-length scaffolds and a highly contiguous genome assembly.

**Results:**

The final draft genome assembly from 33.88 Gb of raw data was ∼397.90 Mb in size, with a 2.05 Mb contig N50. Nine chromosomes were further assembled based on Hi-C data to a 377.19 Mb final size with a 36.26 Mb scaffold N50. The identified repeat sequences accounted for 26.41% of the genome, and 16,006 protein-coding genes were annotated. According to the phylogenetic analysis, *S. miscanthi* is closely related to *Acyrthosiphon pisum*, with *S. miscanthi* diverging from their common ancestor ∼25.0–44.9 million years ago.

**Conclusions:**

We generated a high-quality draft of the *S. miscanthi* genome. This genome assembly should help promote research on the lifestyle and feeding specificity of aphids and their interactions with each other and species at other trophic levels. It can serve as a resource for accelerating genome-assisted improvements in insecticide-resistant management and environmentally safe aphid management.

## Data Description

### Background

**Figure 1.**
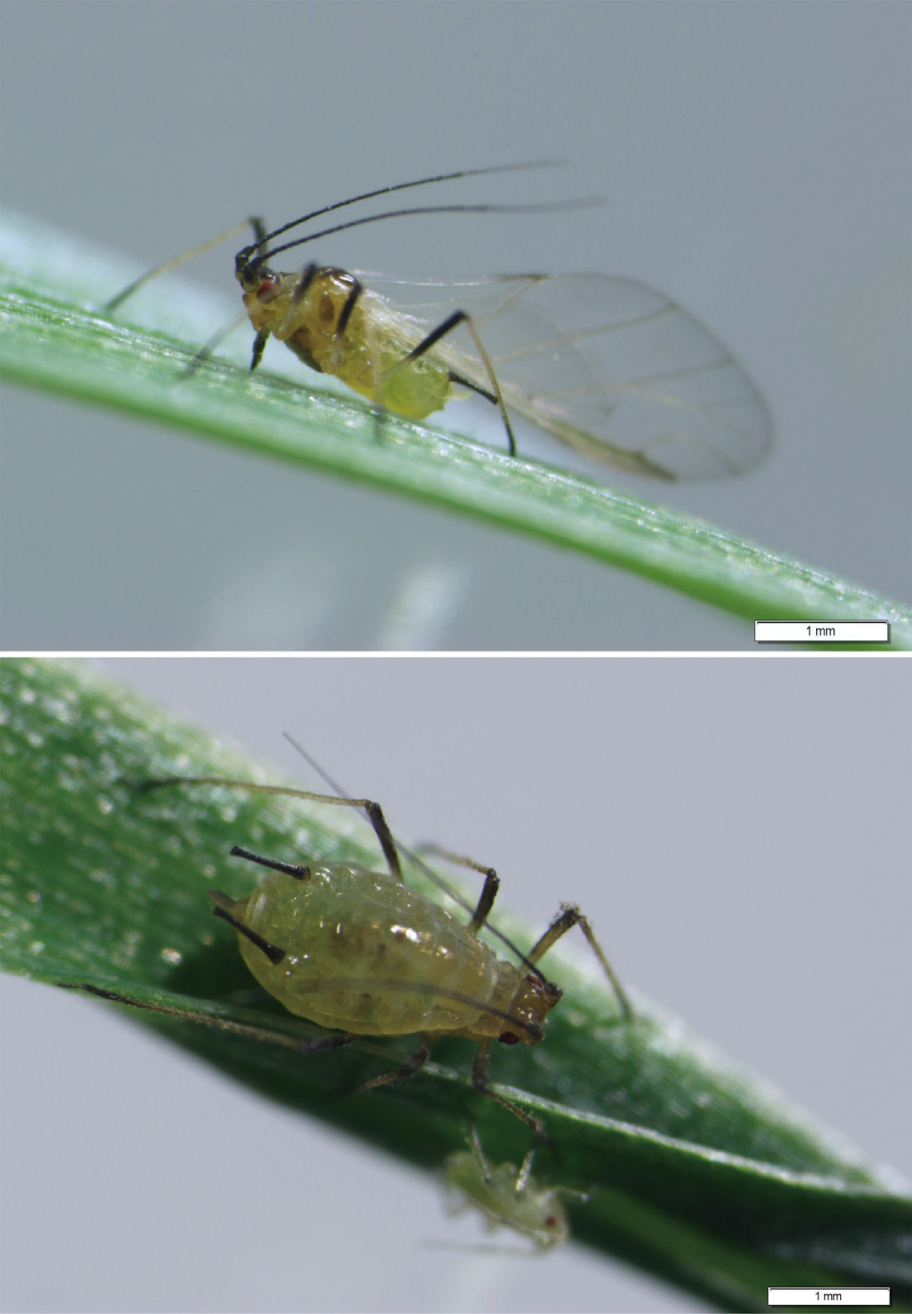
Winged and wingless *S. miscanthi*. Top, winged adult; bottom, wingless adult.

The grain aphid *Sitobion miscanthi* (NCBI:txid44668, Fig. 1), widely mis-reported as *Sitobion avenae* in China [1], is a globally distributed sap-sucking specialist of cereal and a dominant species in wheat-growing regions across China. It threatens wheat production in various ways such as pillaging nutrition from the host, transmitting pathogenic plant viruses, and defecating sticky honeydew that further obstructs photosynthesis and reduces wheat quality. Taken together its highly specialized host range, simple parasitic life cycle, pleomorphism, and alternation of complete and incomplete life cycles make *S. miscanthi* significant for both basic and applied research. Therefore, we sought to publish the genome information for *S. miscanthi*. Genomes with annotation information from 8 aphid species, namely, the pea aphid *Acyrthosiphon pisum* [2], peach aphid *Myzus persicae* [3], soybean aphid *Aphis glycines* [4], Russian wheat aphid *Diuraphis noxia* [5], cherry-oat aphid *Rhopalosiphum padi* [6], black cherry aphid *Myzus cerasi* [6], cotton aphid *Aphis gossypii* [7], and the corn leaf aphid *Rhopalosiphum maidis* [8], are available. However, no genome information for *S. miscanthi* has been published. Here, we report the chromosome-level genome sequence of the *S. miscanthi* isolate Langfang-1, which exhibits higher-quality assembly data indexes than other scaffold-level aphid genomes. Most of the sequences assembled into 9 scaffolds, which supported a 2n = 18 karyotype for *S. miscanthi* [9, 10]. The repeat sequences and phylogenetic relationship of *S. miscanthi* with other insects were further analyzed.

### Sampling

Langfang-1, a grain aphid (*S. miscanthi*) isolate that was originally collected from wheat in Hebei province, was kept in our laboratory for genome sequencing.

An isogenic colony was started from a single parthenogenetic female of *S. miscanthi* and was maintained on wheat (*Triticum aestivum*). Mother aphids were placed into culture dishes (dimeter of 9 cm) with moist absorbent paper on the bottom for 12 h. No newborn nymphs were fed during this period. Newborn nymphs within 12 h without feeding were collected for genome sequencing. In addition, 100 aphids of first and second instars and 50 winged and wingless aphids at the third instar, fourth instar, and adult stages were collected for transcriptome sequencing.

### Genome size estimation

**Figure 2.**
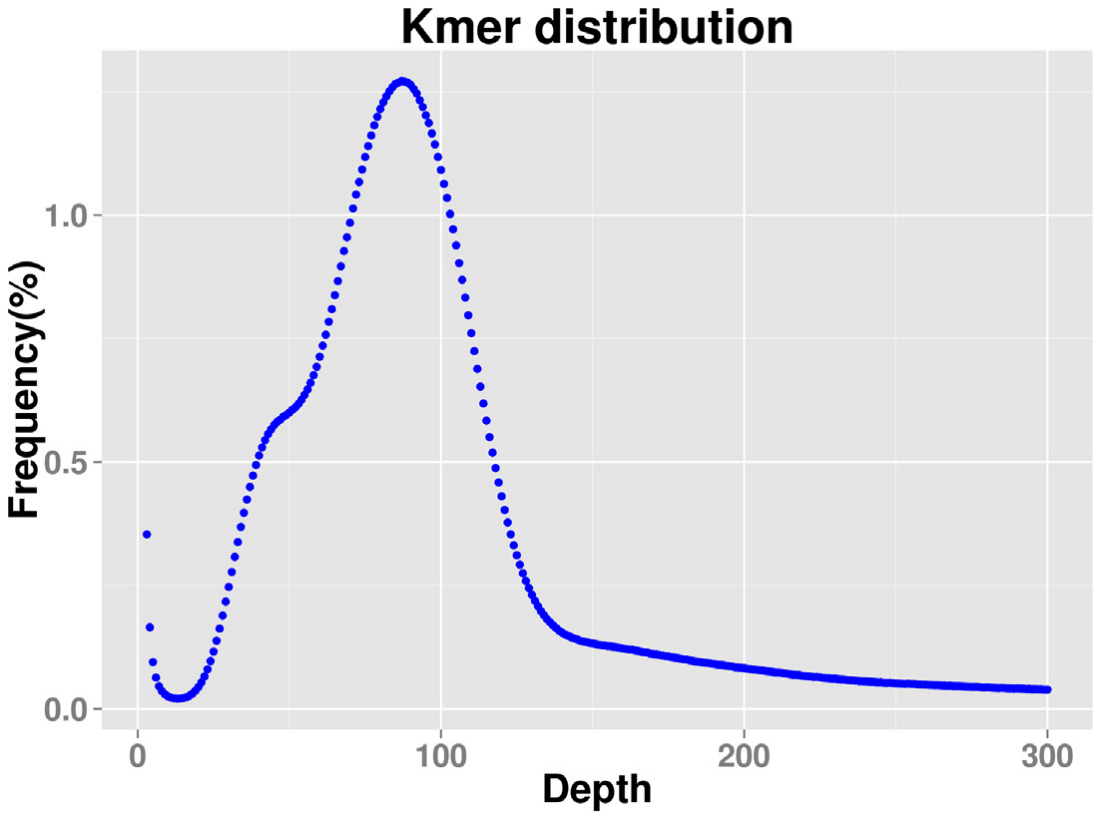
19-mer distribution for the genome size prediction of *S. miscanthi*.

High-quality genomic DNA for sequencing using the Illumina platform (Illumina Inc., San Diego, CA, USA) and PacBio Sequel sequencing (Pacific Biosciences [PacBio], Menlo Park, CA, USA) was extracted from the aforementioned newborn nymphs. The whole-genome size of *S. miscanthi* was estimated by *k*-mer analysis (*k* = 19) based on Illumina DNA sequencing technology [11, 12]. A short-insert library (270 bp) was constructed, and a total of ∼42 Gb of clean reads was obtained for *de novo* assembly to estimate the whole-genome size using the standard protocol provided by the Illumina HiSeq X Ten platform. All clean reads were subjected to 19-mer frequency distribution analysis. The peak of the 19-mer distribution was at a depth of 89, and the genome size of *S. miscanthi* was calculated to be 393.12 Mb (Fig. 2, Table 1).

**Table 1.** Assessment results based on 2 strategies

Genome feature/assessment strategy	19-mer analysis	PacBio
Genome size (Mb)	393.12	397.90
Guanine-cytosine content (%)	31.70	30.25
Repeat sequence content (%)	35.07	24.14
Heterozygosity (%)	0.98	0.57

### Genome assembly using PacBio long reads

The genomic DNA libraries were constructed and sequenced using the PacBio Sequel platform. Additionally, 4.35 million subreads (33.88 Gb in total) with an N50 read length of 12,697 bp were obtained after removing the adapter (Fig. S1).

*De novo* genome assembly with long reads was performed using 2 pipelines, Canu (Canu, RRID:SCR_015880) and wtdbg (WTDBG, RRID:SCR_017225). Because of the high heterozygosity of *S. miscanthi*, in the correction step, Canu first selects longer seed reads with the settings “genomeSize = 400000000” and “corOutCoverage = 50”, then detects overlapping raw reads through the highly sensitive overlapper MHAP (mhap-2.1.2, option “corMhapSensitivity = low/normal/high”), and finally performs an error correction with the falcon_sense method (option “correctedErrorRate = 0.025”). In the next step, with the default parameters, error-corrected reads are trimmed to remove unsupported bases and hairpin adapters to obtain the longest supported range. In the last step, Canu generates the draft assembly using the longest 80 coverage-trimmed reads with Canu v1.5 [13] to output more corrected reads and be more conservative at picking the error rate for the assembly to try to maintain haplotype separation.

Wtdbg is an SMS data assembler that constructs a fuzzy Brujin graph (available at GitHub [14]). Wtdbg first generates a draft assembly with the command “wtdbg -i pbreads.fasta -t 64 -H -k 21 -S 1.02 -e 3 -o wtdbg”. The use of error-corrected reads from Canu results in better assembly performance. Then, a consensus assembly is obtained with the command “wtdbg-cns -t 64 -i wtdbg.ctg.lay -o wtdbg.ctg.lay.fa -k 15”.

To improve genome contiguity, 2 assemblies generated from the Canu and wtdbg pipelines were merged with 2 rounds of quickmerge [15]. Quickmerge uses contigs from wtdbg as query input and contigs from Canu as reference input. The 2 contigs are aligned through mummer (v4.0.0, available at GitHub [16]) with the nucmer parameters “-b 500 -c 100 -l 200 -t 12” and delta-filter parameters “-i 90 -r -q”, and then merged through quickmerge with the parameters “-hco 5.0 -c 1.5 -l 100 000 -ml 5000”. The result was error corrected using Pilon (Pilon, RRID:SCR_014731) [17]. After all of the processing described above, the resulting genome assembly was further cleaned using Illumina next-generation sequencing (NGS) data, which were used in the 19-mer analysis above. The final draft genome assembly was 397.90 Mb, which reached a high level of continuity with a contig N50 length of 2.05 Mb (Table 2). The contig N50 of *S. miscanthi* was much higher than that of previous aphid genome assemblies constructed using DNA NGS sequencing technologies.

**Table 2.** Assembly statistics of the *S. miscanthi* genome and 7 other aphid genomes based mainly on NGS

Genome assembly/species	*S. miscanthi*	*R. padi*	*D. noxia*	*Ac. pisum*	*Ap. glycines*	*M. persicae*	*M. cerasi*	*Ap. gossypii*
Assembly size (Mb)	397.9	319.4	393.0	541.6	302.9	347.3	405.7	294.0
Contig count	1,148	16,689	49,357	60,623	66,000	8,249	56,508	22,569
Contig N50 (bp)	1,638,329	96,831	12,578	28,192	15,844	71,400	17,908	45,572
Scaffold count	656	15,587	5,641	23,924	8,397	4,018	49,286	4,724
Scaffold N50 (bp)	36,263,045	116,185	397,774	518,546	174,505	435,781	23,273	437,960
Genome annotation								
Gene count	16,006	26,286	19,097	36,195	17,558	18,529	28,688	14,694
Mean gene length (kb)	7.805	1,543	1.316	1.964	1.520	1.839	1,222	1.964
Mean exon count per gene	6.7	5.20	3.0	5.0	6.2	6.1	3.7	10.1
Mean exon length (bp)	288	162	249.0	394.7	246	299	178	218

## Genome Quality Evaluation

To assess the completeness of the assembled *S. miscanthi* genome, we subjected the assembled sequences to BUSCO version 2 (BUSCO, RRID:SCR_015008) [18]. Overall, 1,496 and 19 of the 1,658 expected Insecta genes (insect_odb9) were identified in the assembled genome as having complete and partial BUSCO profiles, respectively. Approximately 143 genes were considered missing in our assembly. Among the expected complete Insecta genes, 1,401 and 95 were identified as single-copy and duplicated BUSCOs, respectively (Fig. S4).

### Hi-C library construction and chromosome assembly

In this work, we used Hi-C to further assemble the genome of *S. miscanthi* at the chromosome level. Genomic DNA was extracted for the Hi-C library from the whole aphids of *S. miscanthi* mentioned above. Samples were extracted and sequenced following a standard procedure. Hi-C fragment libraries were constructed with insert sizes of 300–700 bp and sequenced on the Illumina platform. Adapter sequences of raw reads were trimmed, and low-quality paired-end reads were removed for clean data. The clean Hi-C reads were first truncated at the putative Hi-C junctions, and then the resulting trimmed reads were aligned to the assembly results with BWA software (BWA, RRID:SCR_010910) [19]. Only uniquely alignable reads whose mapping quality was >20 were retained for further analysis. Invalid read pairs, including dangling-end and self-cycle, re-ligation, and dumped products, were filtered by HiC-Pro (v2.8.1) [20].

In total, 38.44% of unique mapped read pairs were valid interaction pairs for scaffold correction and were used to cluster, order, and orient scaffolds onto chromosomes by LACHESIS [21].

**Figure 3.**
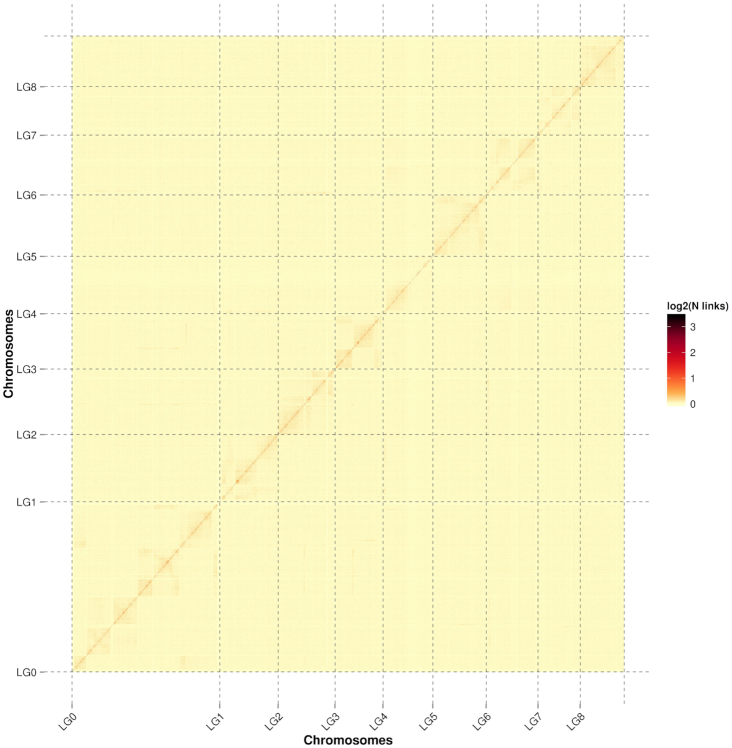
Hi-C contact heat map of the *S. miscanthi* genome.

Before chromosome assembly, we first performed a preassembly for the error correction of scaffolds, which required the splitting of scaffolds into segments of 50 kb on average. The Hi-C data were mapped to these segments using BWA (version 0.7.10-r789) software. The uniquely mapped data were retained to perform assembly by using LACHESIS software. Any 2 segments that showed inconsistent connection with information from the raw scaffold were checked manually. These corrected scaffolds were then assembled with LACHESIS. Parameters for running LACHESIS included CLUSTER_MIN_RE_SITES, 70; CLUSTER_MAX_LINK_DENSITY, 1; ORDER_MIN_N_RES_IN_TRUN, 19; ORDER_MIN_N_RES_IN_SHREDS, 19. After this step, placement and orientation errors exhibiting obvious discrete chromatin interaction patterns were manually adjusted. Finally, 774 scaffolds (representing 97.48% of the total length) were anchored to 9 chromosomes (Fig. 3, Table S1). A genome with a final size of 377.19 Mb and a scaffold N50 of 36.26 Mb was assembled, which showed a high level of continuity with a contig N50 of 2.05 Mb using 1,167 contigs. The contig N50 size of the genome assembled using PacBio long reads and Hi-C assembly was much higher than that of the 7 previously published aphid genome assemblies constructed using DNA NGS technologies (Table 3).

**Table 3.** Summary of *S. miscanthi* genome assembly

Statistics	Draft scaffolds	Corrected by Hi-C
Contig number	1,039	1,167
Contig length	397,907,165	397,907,165
Contig N50 (bp)	2,049,770	1,565,814
Contig N90 (bp)	256,083	185,510
Contig max (bp)	11,219,273	10,100,000
Gap number/gap total length (bp)	0	0

### Repeat sequences within the *S. miscanthi* genome assembly

To identify tandem repeats, and based on the classification of eukaryotic transposable elements (TE) by Wicker et al. [22], we utilized 4 software programs, namely, LTR_FINDER (v1.0.5; LTR_Finder, RRID:SCR_015247) [23], MITE-Hunter (v1.0.0) [24], RepeatScout (v1.0.5; RepeatScout, RRID:SCR_014653) [25], and PILER-DF (v1.0) [26], to build a *de novo* repeat library based on our assembly with the default settings. Subsequently, the predicted repeats were classified using PASTEClassifier (v1.0) [27] and merged with Repbase (19.06) [28]. Finally, using the resulting repeat database as the final repeat library, RepeatMasker v4.0.5 (RepeatMasker, RRID:SCR_012954) [29] was used to identify repetitive sequences in the *S. miscanthi*genome with the following parameters: “-nolow -no is -norna -engine wublast”. The repeat sequences accounted for 31.15% of the *S. miscanthi* genome, including identified repeat sequences (26.42% of the genome), based on the *de novo*repeat library (Table 4).

**Table 4.** Detailed classification of repeats in the *S. miscanthi* genome assembly

Type	Number	Length (bp)	Rate (%)
Class I (Retrotransposons)	194,093	51,169,345	12.86
DIRS (Dictyostelium intermediate repeat sequence)	1,289	695,762	0.17
LINE (Long interspersed nuclear element)	40,230	10,832,765	2.72
LTR (Long terminal repeats) /Copia	2,438	742,051	0.19
LTR/Gypsy	18,807	6,949,790	1.75
LTR/Unknown	7,534	3,195,404	0.8
PLE (Penelope-like elements)|LARD (Large retrotransposon derivatives)	115,765	28,920,417	7.27
SINE (Short interspersed nuclear element)	6,665	1,075,456	0.27
SINE|TRIM	15	5,478	0
TRIM (Terminal repeat retrotransposons in miniature)	1,116	1,281,655	0.32
Class I Unknown	234	26,384	0.01
Class II (DNA transposons)	188,820	44,184,063	11.1
Crypton	299	20,282	0.01
Helitron	5,688	1,871,785	0.47
MITE (Miniature inverted repeat transposable elements)	7,972	1,434,924	0.36
Maverick	7,888	3,289,168	0.83
TIR (Terminal inverted repeat)	89,268	22,913,523	5.76
Class II unknown	77,705	15,793,696	3.97
Potential host gene	926	251,812	0.06
SSR (Simple sequence repeats)	2,611	381,142	0.1
Unknown	74,204	18,832,522	4.73
Identified	386,450	105,110,753	26.42
Total	460,654	123,943,275	31.15

### Transcriptome sequencing to aid in gene prediction

Transcriptome sequencing (Illumina RNA-Seq and PacBio Iso-Seq) of complementary DNA (cDNA) libraries prepared from the whole newborn nymphs of *S. miscanthi* was conducted to aid in gene prediction. High-quality RNA was extracted using an SV Total RNA isolation kit (Promega, Madison, WI, USA). Reverse transcription was completed using a Clontech SMARTer cDNA synthesis kit (Clontech Laboratories, Palo Alto, CA, USA). A paired-end library was then prepared following the Paired-End Sample Preparation Kit manual (Illumina). Finally, a library with an insert length of 300 bp was sequenced by an Illumina HiSeq X Ten in 150PE mode (Illumina). As a result, we obtained ∼8.707 Gb of transcriptome data from RNA-seq. The quality of the transcripts was assessed by the proportion of gene regions covered by these transcripts, higher being better. In this case, the proportion was 85.66%. The assembled transcripts were used to improve predictions of protein-coding genes in the *S. miscanthi* genome.

### Gene annotation

Gene prediction of the *S. miscanthi* genome was performed using *de novo*, homology-based and transcriptome sequencing-based predictions. For *de novo* prediction, we used Augustus v2.4 (Augustus, RRID:SCR_008417) [30], GlimmerHMM v3.0.4 (GlimmerHMM, RRID:SCR_002654) [31], SNAP (version 2006–07-28; SNAP, RRID:SCR_007936) [32], GeneID v1.4 [33], and GENSCAN (GENSCAN, RRID:SCR_012902) [34] software to predict protein-coding genes in the *S. miscanthi* genome assembly. For homology-based prediction, protein sequences of closely related aphid species, namely, *Sipha flava, D. noxia, A. pisum*, and *M. persicae*, were aligned against the *S. miscanthi* genome to predict potential gene structures using GeMoMa v1.3.1 [35]. For transcriptome sequencing-based prediction, we assembled the NGS transcriptome short reads into unigenes without a reference genome and then predicted genes based on unigenes using PASA v2.0.2 (PASA, RRID:SCR_014656) [36]. All of the above gene models were then integrated using EVM v1.1.1 [37] to obtain a consensus gene set. The final total gene set for the *S. miscanthi* genome was composed of 16,006 genes with an average of 6.74 exons per gene. The gene number, gene length distribution, and exon length distribution were all comparable to those of other aphid species (Table 2). Moreover, the indexes such as contig count and scaffold count were much improved.

To obtain further functional annotation of the protein-coding genes in the *S. miscanthi* genome, we used BLAST v2.2.31 [38] to align the predicted genes with functional databases such as the nonredundant protein (NR) [39], EuKaryotic Orthologous Groups (KOG) [40], Gene Ontology (GO) [41], KEGG [42], and Translation of European Molecular Biology Laboratory (TrEMBL) [43] databases (e-value ≤ 1e^−5^) (Figs S2 and S3). Ultimately, 99.35% (15,902 genes) of the 16,006 genes were annotated based on ≥1 database (Table S2).

### Gene family identification and phylogenetic tree construction

**Figure 4.**
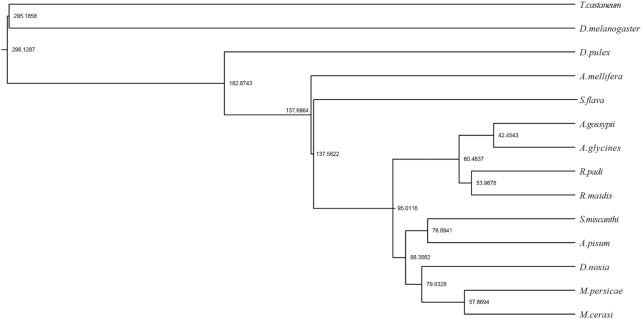
The phylogenetic relationships of *S. miscanthi* with other arthropods.

We used the OrthoMCL program [44] with an e-value threshold of 1e−5 to identify gene families based on the protein alignments of each gene from *S. miscanthi* and those of other insect species, which included *R. padi, D. noxia, A. pisum, M. persicae, A. glycines, M. cerasi, R. maidis, A. gossypii, S. flava* [45], *Apis mellifera* [46], *D. pulex* [47], *Drosophila melanogaster* [48], and *Tribolium castaneum* [49]. A total of 14,722 genes were identified by clustering the homologous gene sequences from 10,918 gene families (Fig. S4). One hundred thirty-eight gene families were specific to *S. miscanthi*. Subsequently, we selected 2,605 single-copy orthogroups from the abovementioned species to reconstruct the phylogenetic relationships between *S. miscanthi* and other arthropod species. A phylogenetic tree was constructed with the maximum-likelihood method implemented in the PhyML package [50]. We used the MCMCTree program to estimate divergence times among species based on the approximate likelihood method [51] and with molecular clock data for the divergence time of medaka from the TimeTree database [52]. According to the phylogenetic analysis, *S. miscanthi* clustered with *A. pisum*. The divergence time between *S. miscanthi* and its common ancestor shared with *A. pisum* was ∼76.8–88.4 million years (Fig. 4).

## Conclusions

1. We successfully assembled the chromosome-level genome of *S. miscanthi* based on long reads from the third-generation PacBio Sequel sequencing platform.
2. The size of the final draft genome assembly was ∼397.90 Mb, which was slightly larger than the estimated genome size (393.12 Mb) based on *k*-mer analysis. The contigs were scaffolded onto chromosomes using Hi-C data with a contig N50 of 2.05 Mb and a scaffold N50 of 36.26 Mb. We also predicted 16,006 protein-coding genes from the generated assembly, and 99.35% (15,902 genes) of all protein-coding genes were annotated.
3. We found that the divergence time between *S. miscanthi* and its common ancestor shared with *A. pisum* was ∼76.8–88.4 million years.
4. The assembly of this genome will help promote research on the lifestyle and feeding specificity of aphids as well as their interactions with each other and other trophic levels and can serve as a resource for accelerating genome-assisted improvements in insecticide-resistant management as well as environmentally safe aphid management.

## Availability of supporting data and materials

Data supporting the results of this article have been deposited at DDBJ/ENA/GenBank under Bioproject PRJNA532495 and the accession SSSL00000000. The version described in this article is version SSSL01000000. Other supporting data and materials including annotations and phylogenetic trees are available in the *GigaScience* GigaDB database [53].

## Additional files

**Figure S1:** Filtered subread length distribution

**Figure S2:** KOG annotation result

**Figure S3:** KEGG annotation result

**Figure S4:** Statistics of gene family clusters

**Table S1:** Summary of genome constructed to chromosome level of *S. avenae*

**Table S2:** genome annotation

giz101_GIGA-D-19-00137_Original_Submission

giz101_GIGA-D-19-00137_Revision_1

giz101_GIGA-D-19-00137_Revision_2

giz101_Response_to_Reviewer_Comments_Original_Submission

giz101_Response_to_Reviewer_Comments_Revision_1

giz101_Reviewer_1_Report_Original_Submission Andrew Michel -- 5/20/2019 Reviewed

giz101_Reviewer_1_Report_Revision_1 Andrew Michel -- 7/16/2019 Reviewed

giz101_Reviewer_2_Report_Original_Submission Anna-Maria Botha -- 6/5/2019 Reviewed

giz101_Supplemental_File

## Abbreviations

BLAST: Basic Local Alignment Search Tool; bp: base pairs; BUSCO: Benchmarking Universal Single-Copy Orthologs; BWA: Burrows-Wheeler Aligner; cDNA: complementary DNA; EVM: EVidenceModeler; Gb: gigabase pairs; GeMoMa: Gene Model Mapper; GO: Gene Ontology; kb: kilobase pairs; KEGG: Kyoto Encyclopedia of Genes and Genomes; LINE: long interspersed nuclear element; KOG: EuKaryotic Orthologous Groups; LTR: long terminal repeat; Mb: megabase pairs; MHAP: MinHash Alignment Process; MITE: miniature inverted-repeat transposable element; NCBI: National Center for Biotechnology Information; NGS: next-generation sequencing; NR: Nonredundant protein; NT: Nonredundant nucleotide; PacBio: Pacific Biosciences; PASA: Program to Assemble Spliced Alignments; SINE: short interspersed nuclear element; TrEMBL: Translation of European Molecular Biology Laboratory.

## Competing interests

The authors declare that they have no competing interests.

## Funding

This research was sponsored by the National Key R & D Plan of China (Nos. 2017YFD0200900, 2016YFD0300700, and 2017YFD0201700), the National Natural Science Foundation of China (Nos. 31871966 and 31871979), China Postdoctoral Science Foundation (No. 2018M631646), the State Modern Agricultural Industry Technology System (No. CARS-22-G-18), and the China Scholarship Council (No. 201703250048).

## Authors' contributions

J.F. and J.C. conceived the project; X.J. and Q.Z. raised the aphids; X.J. and Y.Q. collected the samples for both genome and transcriptome sequencing; Q.Z., X.J., and J.F. isolated the genomic DNA for both the 19-mer analysis and genome sequencing; J.F., Q.Z., and S.Z. isolated the total RNA for transcriptome sequencing; J.F. and H.Y. performed the genome as well as transcriptome assembly, annotated the genome, and conducted other data analysis; Q.L. and Y.Z. took the photographs of *S. miscanthi*; and J.F. and H.Y. wrote the manuscript.
