# Supplementary material for: A chromosome-level draft genome of the grain aphid Sitobion miscanthi
Source: Gigascience. 2019 Aug 20;8(8):giz101. doi: 10.1093/gigascience/giz101 (PMC6701489; doi:10.1093/gigascience/giz101)
Supplement: giz101_GIGA-D-19-00137_Original_Submission [file giz101_giga-d-19-00137_original_submission.pdf]

GigaScience

A chromosome-level draft genome of the grain aphid Sitobion miscanthi

--Manuscript Draft--

**Manuscript Number:**	GIGA-D-19-00137
**Full Title:**	A chromosome-level draft genome of the grain aphid Sitobion miscanthi
**Article Type:**	Data Note
**Funding Information:**	National Key R & D Plan of China (2017YFD0200900) — Prof. Julian Chen<br>National Key R & D Plan of China (2016YFD0300700) — Dr. Jia Fan<br>National Key R & D Plan of China (2017YFD0201700) — Dr. Jia Fan<br>National Natural Science Foundation of China (31871966) — Dr. Jia Fan<br>National Natural Science Foundation of China (31371946) — Prof. Julian Chen<br>State Modern Agricultural Industry Technology System (CARS-22-G-18) — Dr. Jia Fan<br>China Scholarship Council (201703250048) — Dr. Jia Fan
**Abstract:**	Background: Sitobion miscanthi is a wheat sap-sucking pest worldwide, and an ideal model for studies of host plant specificity, parthenogenesis-based phenotypic plasticity, and interactions between insects and other species of various trophic levels, such as viruses, bacteria, plants and natural enemies. This species was previously misidentified in China as Sitobion avenae. We identified it as S. miscanthi and analyzed the entire genome of a female aphid colony using long-read sequencing and Hi-C data to generate chromosome-length scaffolds and a highly contiguous genome assembly.<br>Results<br>1.All aphids originally collected from 18 different geographical regions across China had morphological traits consistent with those of S. miscanthi.<br>2.The final draft genome assembly from 33.88 Gb of raw data was approximately 397.90 Mb with a 2.05 Mb contig N50. Nine chromosomes were further assembled based on Hi-C data to a 377.19 Mb final size with a 36.26 Mb scaffold N50.<br>3.The identified repeat sequences accounted for 26.41% of the genome, and 16,006 protein-coding genes were annotated. According to the phylogenetic analysis, S. miscanthi is closely related to Acyrthosiphon pisum, with S. miscanthi diverging from their common ancestor approximately 25.0-44.9 million years ago.<br>Conclusions: We identified S. miscanthi and generated a high-quality draft of its genome. This genome assembly promotes research on the lifestyle and feeding specificity of aphids and their interactions with each other and species at other trophic levels and can serve as a resource for accelerating genome-assisted improvements in chemical drug resistant management and environmentally friendly aphid management.<br>Keywords: aphid, Sitobion miscanthi, Sitobion avenae, annotation, genome, long-read sequencing, Hi-C assembly
**Corresponding Author:**	Jia Fan<br><br>CHINA
**Corresponding Author Secondary Information:**	
**Corresponding Author's Institution:**	
**Corresponding Author's Secondary Institution:**	
**First Author:**	Xin Jiang

Powered by Editorial Manager® and ProduXion Manager® from Aries Systems Corporation

First Author Secondary Information:	
Order of Authors:	Xin Jiang
	Qian Zhang
	Yaoguo Qin
	Siyu Zhang
	Qian Li
	Yong Zhang
	Hang Yin
	Jia Fan
	Julian Chen
Order of Authors Secondary Information:	
Additional Information:	
**Question**	**Response**
Are you submitting this manuscript to a special series or article collection?	No
**Experimental design and statistics**<br><br>Full details of the experimental design and statistical methods used should be given in the Methods section, as detailed in our Minimum Standards Reporting Checklist. Information essential to interpreting the data presented should be made available in the figure legends.<br><br>Have you included all the information requested in your manuscript?	Yes
**Resources**<br><br>A description of all resources used, including antibodies, cell lines, animals and software tools, with enough information to allow them to be uniquely identified, should be included in the Methods section. Authors are strongly encouraged to cite Research Resource Identifiers (RRIDs) for antibodies, model organisms and tools, where possible.<br><br>Have you included the information requested as detailed in our Minimum Standards Reporting Checklist?	Yes

**Availability of data and materials**<br><br>All datasets and code on which the conclusions of the paper rely must be either included in your submission or deposited in publicly available repositories (where available and ethically appropriate), referencing such data using a unique identifier in the references and in the “Availability of Data and Materials” section of your manuscript.<br><br>Have you have met the above requirement as detailed in our Minimum Standards Reporting Checklist?	Yes

Manuscript

Click here to download Manuscript Genome of Smiscanthi.docx

A chromosome-level draft genome of the grain aphid *Sitobion miscanthi*

Xin Jiang*, Qian Zhang*, Yaoguo Qin*, Siyu Zhang, Qian Li, Yong Zhang, Hang Yin, Jia Fan†, Julian Chen†

Affiliations: All authors, Institute of Chinese Academy of Agricultural Sciences, 2 Yuanmingyuan West Road, Haidian District, Beijing, 100193, China. Jia Fan, Tel: +86-01062815934, E-mail: jfan@ippcaas.cn; Julian Chen, 86-10-62813685, E-mail: jlchen@ippcaas.cn; Xin Jiang, Tel: +86-01062815934; E-mail: 18911895763@163.com; Qian Zhang, Tel: +86-01062815934; E-mail: zhangqianelaine@163.com; Yaoguo Qin, Tel: +86-01062815934; E-mail: qinyg1018@163.com; Siyu Zhang, Tel: +86-01062815934; E-mail: Zhangsiyu1567@163.com; Qian Li, Tel: +86-01062815934; E-mail: liqian0927@yeah.net; Yong Zhang, Tel: +86-01062815934; E-mail: zhangyongnky@163.com; Hang Yin, Tel: +86-01062815934; E-mail: yhang01@163.com;

†**Corresponding authors:** Jia Fan, Institute of Chinese Academy of Agricultural Sciences, 2 Yuanmingyuan West Road, Haidian District, Beijing, 100193, P. R., China, 86-10-62815934, jfan@ippcaas.cn.

Julian Chen, Institute of Chinese Academy of Agricultural Sciences, 2 Yuanmingyuan West Road, Haidian District, Beijing, 100193, P. R., China, 86-10-62813685, jlchen@ippcaas.cn.

*Contributed equally to this work

Abstract

Background**:** *Sitobion miscanthi* is a wheat sap-sucking pest worldwide, and an ideal model for studies of host plant specificity, parthenogenesis-based phenotypic plasticity, and interactions between insects and other species of various trophic levels, such as viruses, bacteria, plants and natural enemies. This species was previously misidentified in China as *Sitobion avenae*. We identified it as *S. miscanthi* and analyzed the entire genome of a female aphid colony using long-read sequencing and Hi-C data to generate chromosome-length scaffolds and a highly contiguous genome assembly.

Results

1. All aphids originally collected from 18 different geographical regions across China had morphological traits consistent with those of *S. miscanthi*.
2. The final draft genome assembly from 33.88 Gb of raw data was approximately 397.90 Mb with a 2.05 Mb contig N50. Nine chromosomes were further assembled based on Hi-C data to a 377.19 Mb final size with a 36.26 Mb scaffold N50.
3. The identified repeat sequences accounted for 26.41% of the genome, and 16,006 protein-coding genes were annotated. According to the phylogenetic analysis, *S. miscanthi* is closely related to *Acyrthosiphon pisum*, with *S. miscanthi* diverging from their common ancestor approximately 25.0-44.9 million years ago.

Conclusions**:** We identified *S. miscanthi* and generated a high-quality draft of its genome. This genome assembly promotes research on the lifestyle and feeding specificity of aphids and their interactions with each other and species at other trophic levels and can serve as a resource for accelerating genome-assisted improvements in chemical drug resistant management and environmentally friendly aphid management.

Keywords: aphid, *Sitobion miscanthi*, *Sitobion avenae*, annotation, genome, long-read sequencing, Hi-C assembly

Data Description

Background

The grain aphid *Sitobion miscanthi* (Figure1), a cereal specialist, is a globally distributed sap-sucking pest of wheat and a dominant species in wheat-growing regions across China. It threatens wheat production in various ways such as pillaging nutrition from the host, transmitting pathogenic plant viruses, and defecating sticky honeydew that further obstructs photosynthesis and reduces wheat product quality. Together with its highly specialized host scope, the simple parasitic life cycle, pleomorphism, and alternation of complete and incomplete life cycles make *S. miscanthi* an ideal model insect for both basic and applied research. This species was misidentified [1] as *Sitobion avenae* in China. Because morphological characteristics are inconclusive for identification purposes [2], we sought to publish the genome information for *S. miscanthi* here. Published genomes with annotation information of 6 aphid species (the pea aphid *Acyrthosiphon pisum* [3], peach aphid *Myzus persicae* [4], soybean aphid *Aphis glycines* [5], Russian wheat aphid *Diuraphis noxia* [6], cherry-oat aphid, *Rhopalosiphum padi* [7], and black cherry aphid *Myzus cerasi* [7]) are available on the website https://bipaa.genouest.org/is/aphidbase/, the genome of the cotton aphid Aphis gossypii [8] is available at http://119.78.67.200/Agos_IBMB.html, and the genome of the corn leaf aphid *Rhopalosiphum maidis* [9] is available at https://www.ncbi.nlm.nih.gov/genome/?term=txid43146[orgn]. However, no genome information for this species has been published. Here, we report the chromosome-level genome sequence of the *S. miscanthi* isolate Langfang-1, which exhibits higher-quality assembly data indexes than other scaffold-level of aphid genomes. Most of the sequences assembled into 9 scaffolds, which supported a 2n=18 karyotype for *S. miscanthi*. The repeat

sequences and phylogenetic relationship of *S. miscanthi* with other insects were further analyzed.

Sampling

Langfang-1, a grain aphid (*S. miscanthi*) isolate that was originally collected from wheat in Hebei province, was kept in our laboratory for morphological observation and genome sequencing. In addition, other populations of *S. miscanthi* originally collected from 17 different wheat-growing regions of China were also morphologically observed for species identification.

An isogenic colony was started from a single parthenogenetic female of *S. miscanthi* and was maintained on wheat (*Triticum aestivum*). Newborn nymphs within 12 h without feeding were collected for genome sequencing. In addition, 100 aphids of 1st and 2nd instars and 50 winged and wingless aphids at the 3rd instar, 4th instar and adult stages were collected for transcriptome sequencing.

Morphological observation of S. miscanthi

Morphological observations were performed on an ultradepth three-dimensional microscope (VHX-2000C, KEYENCE, OSA, Japan). We focused on the antennae of winged aphids as well as cauda and cornicles of both winged and wingless aphids, which are the most distinct structures between *S. avenae* and *S. miscanthi* [2]. Langfang-1 as well as populations from another 17 areas across China (Figure S1) exhibited consistent morphologies (Figure 2): 1) secondary rhinaria in a line and generally distributed on 2/3 of the third segment near the base;

2) cornicle at least 1.4 times longer than cauda; and 3) 6-8 hairs inserted into the cauda. These characters are consistent with the morphological keys of *S. miscanthi*. Therefore, we concluded that the dominant wheat aphid in China is *S. miscanthi*.

Genome size estimation

High-quality genomic DNA for sequencing using the Illumina platform (Illumina Inc., San Diego, CA, USA) and PacBio Sequel sequencing (Pacific Biosciences of California, Menlo Park, CA, USA) was extracted from the newborn nymphs mentioned above. The whole-genome size of *S. miscanthi* was estimated by *k*-mer analysis (*k*=19) based on Illumina DNA sequencing technology. A short-insert library (270 bp) was constructed, and a total of ~42 Gb of clean reads was finally obtained for de novo assembly to estimate the whole-genome size using the standard protocol provided by the Illumina HiSeq X Ten platform. All clean reads were subjected to 19-mer frequency distribution analysis. The peak of 19-mers was at a depth of 89, and the genome size of *S. miscanthi* was calculated to be 393.1 Mb (Figure 3, Table 1).

Genome assembly using PacBio long reads

The genomic DNA libraries were constructed and sequenced using the PacBio Sequel platform. Additionally, 4.35 million subreads (33.88 Gb in total) with an N50 read length of 12,697 bp were obtained after removing the adaptor (Figure S2). Canu v1.4 [10] was first used to correct clean data and assemble the genome. A genome assembly with a total length of 585.29 Mb was constructed for *S. miscanthi*, which was larger than the genome size estimated by 19-mer analysis. We also applied wtdbg (https://github.com/ruanjue/wtdbg) to obtain a genome assembly size of 392.59 Mb based on the corrected clean data. Finally, both genome results were merged using Quickmerge [11] and error correction was implemented using Pilon [12]. The resulting genome assembly was further cleaned using Illumina NGS

data, which were used in the 19-mer analysis above. The final draft genome assembly was 397.90 Mb, which reached a high level of continuity with a contig N50 length of 2.05 Mb (Table 2). The contig N50 of *S. miscanthi* was much higher than that of previous aphid genome assemblies constructed using DNA NGS sequencing technologies.

Genome quality evaluation

To assess the completeness of the assembled *S. miscanthi* genome, we subjected the assembled sequences to Benchmarking Universal Single-Copy Orthologs (BUSCO) version 2 [13]. Overall, 1496 and 19 of the 1658 expected Insecta genes (insect_odb9) were identified in the assembled genome as having complete and partial BUSCO profiles, respectively. Approximately 143 genes were considered missing in our assembly. Among the expected complete Insecta genes, 1401 and 95 were identified as single copy and duplicated BUSCOs, respectively (Table S1).

Hi-C library construction and chromosome assembly

In this work, we used Hi-C to further assemble the genome of *S. miscanthi* at the chromosome level. Genomic DNA was extracted for the Hi-C library from the whole aphids of *S. miscanthi* mentioned above. Samples were extracted and sequenced following a standard procedure [e.g., [14, 15]]. One hundred and two million 150-bp paired-end Illumina reads (30.65 Gb) with a Q30 of at least 90.03% were produced. By mapping the Hi-C data to the PacBio-based assembly using BWA software, we obtained sequencing data with mates mapped to a different contig, and the amounts of data mapped to a different contig were 166.6 Mb (81.46%) and 57.63 Mb (56.34%), respectively. We then employed Lachesis software to correct the constructed contigs based on the merge strategy and aligned paired-end reads. We obtained 1,167 cleaned contigs by interrupting misassembly of 1,039 contigs. According to the clustering, ordering, and orienting of the contigs (1,039) to the

assembly, these sequences were grouped into 9 chromosome clusters and scaffolded using Lachesis software; the scaffolds corresponded to the 9 chromosomes of *S. miscanthi* based on the karyotype analyses (Figure 4, Table S2). A genome with a final size of 377.19 Mb, accounting for 97.24% of the draft genome, and a scaffold N50 of 36.26 Mb was assembled, which showed a high level of continuity with a contig N50 of 2.05 Mb using 1,167 contigs. The anchor rate of contigs to chromosomes was as high as 97.24% based on the Hi-C assembly. The contig N50 of the genome assembled using PacBio long reads and Hi-C assembly was much higher than that of the 7 previously published aphid genome assemblies constructed using DNA NGS technologies (Table 3).

Repeat sequences within the S. miscanthi genome assembly

To identify tandem repeats, we utilized Tandem Repeat Finder to annotate repetitive elements in the *S. miscanthi* genome. LTR_FINDER [16], MITE-Hunter [17], RepeatScout v1.0.5 [18], and PILER-DF v2.4 [19] were used with the default parameters to construct a de novo repeat library. Subsequently, we used RepeatMasker v4.06 [20] to map our assembled sequences to Repbase [21] and the de novo repeat library, which was classified using PASTEClasifier [22] to predict repeat sequences. The repeat sequences accounted for 31.15% of the *S. miscanthi* genome, including identified repeat sequences (26.42% of the genome), based on the de novo repeat library (Table 4).

Transcriptome sequencing to aid in gene prediction

Transcriptome sequencing (Illumina RNA-Seq and PacBio Iso-Seq) of cDNA libraries prepared from the whole newborn nymphs of *S. miscanthi* was conducted to aid in gene

prediction. High-quality RNA was extracted using an SV Total RNA isolation kit (Promega, Madison, WI, USA). Reverse transcription was completed using a Clontech SMARTer cDNA synthesis kit (Clontech Laboratories, Palo Alto, CA, USA). A paired-end library was then prepared following the Paired-End Sample Preparation Kit manual (Illumina Inc., San Diego, CA, USA). Finally, a library with an insert length of 300 bp was sequenced by an Illumina HiSeq X Ten in 150PE mode (Illumina Inc., San Diego, CA, USA). As a result, we obtained ~8.707 Gb of high-quality transcriptome data from RNA-seq. The assembled transcripts were used to improve predictions of protein-coding genes in the *S. miscanthi* genome.

Gene annotation

Gene prediction of the *S. miscanthi* genome was performed using de novo, homology-based and transcriptome sequencing-based predictions. For de novo prediction, we employed Augustus v2.4 [23], GlimmerHMM v3.0.4 [24], SNAP (version 2006-07-28) [25], GeneID v1.4 [26] and GENSCAN [27] software to predict protein-coding genes in the *S. miscanthi* genome assembly. For homology-based prediction, protein sequences of closely related aphid species, namely, *Sipha flava*, *D. noxia*, *Ac. pisum* and *M. persicae*, were aligned against the *S. miscanthi* genome to predict potential gene structures using GeMoMa v1.3.1 [28]. For transcriptome sequencing-based prediction, we assembled the NGS transcriptome short reads into unigenes without a reference genome and then predicted genes based on unigenes using PASA v2.0.2 [29]. All of the above gene models were then integrated using EVM v1.1.1 [30] to obtain a consensus gene set. The final total gene set for the *S. miscanthi* genome was composed of 16,006 genes with an average of 6.74 exons per gene. The gene number, gene

length distribution, and exon length distribution were all comparable with those of other aphid species (Table 1). Moreover, the indexes such as contig count and scaffold count were much improved.

To obtain further functional annotation of the protein-coding genes in the *S. miscanthi* genome, we employed the BLAST v2.2.31 [31] program to align the predicted genes with functional databases such as the nonredundant protein (NR) [32], EuKaryotic Orthologous Groups (KOG) [33], Gene Ontology (GO) [34], Kyoto Encyclopedia of Genes and Genomes (KEGG) [35], and Translation of European Molecular Biology Laboratory (TrEMBL) [36] databases (e-value $\leqslant 1e^{-5}$) (Figures S2 and 3). Ultimately, 99.35% (15,902 genes) of the 16,006 genes were annotated based on at least one database (Table S3).

Gene family identification and phylogenetic tree construction

We employed the OrthoMCL program [37] with an e-value threshold of 1e-5 to identify gene families based on the protein alignments of each gene from *S. miscanthi* and those of other insect species, which included *R. padi*, *D. noxia*, *Ac. pisum*, *M. persicae*, *Ap. glycines*, *M. cerasi*, *Rhopalosiphum maidis*, *Ap. gossypii*, *S. flava* (ftp://ftp.ncbi.nlm.nih.gov/genomes/all/GCF/003/268/045/GCF_003268045.1_YSA_version1/GCF_003268045.1_YSA_version1_genomic.fna.gz), *Apis mellifera* (ftp://ftp.ncbi.nlm.nih.gov/genomes/all/GCF/003/254/395/GCF_003254395.2_Amel_HAv3.1/GCF_003254395.2_Amel_HAv3.1_genomic.fna.gz), *D. pulex* (ftp://ftp.ncbi.nlm.nih.gov/genomes/all/GCA/000/187/875/GCA_000187875.1_V1.0/GCA_000187875.1_V1.0_genomic.fna.gz), *Drosophila melanogaster*

(ftp://ftp.ncbi.nlm.nih.gov/genomes/all/GCF/000/001/215/GCF_000001215.4_Release_6_plus_ISO1_MT/GCF_000001215.4_Release_6_plus_ISO1_MT_genomic.fna.gz) and *Tribolium castaneum* (ftp://ftp.ncbi.nlm.nih.gov/genomes/all/GCF/000/002/335/GCF_000002335.3_Tcas5.2/GCF_000002335.3_Tcas5.2_genomic.fna.gz). A total of 14,722 genes were identified by clustering the homologous gene sequences from 10,918 gene families (Figure S5). One hundred thirty-eight gene families were specific to *S. miscanthi*. Subsequently, we selected 2,605 single-copy orthogroups from the abovementioned species to reconstruct the phylogenetic relationships between *S. miscanthi* and other arthropod species. A phylogenetic tree was constructed with the maximum-likelihood method implemented in the PhyML package [38]. We used the MCMCTree program to estimate divergence times among species based on the approximate likelihood method [39] and with molecular clock data for the divergence time of medaka from the TimeTree database [40]. According to the phylogenetic analysis, *S. miscanthi* clustered with *Ac. pisum*. The divergence time between *S. miscanthi* and its common ancestor shared with *Ac. pisum* was approximately 76.8-88.4 million years (Figure5).

Conclusions

1. The dominant species of wheat aphid in China is *S. miscanthi* instead of *S. avenae*.
2. We successfully assembled the chromosome-level genome of *S. miscanthi* based on long reads from the third-generation PacBio Sequel sequencing platform.

The size of the final draft genome assembly was approximately 397.91 Mb, which was slightly larger than the estimated genome size (393.12 Mb) based on *k*-mer analysis. The contigs were scaffolded onto chromosomes using Hi-C data with a

contig N50 of 2.05 Mb and a scaffold N50 of 36.26 Mb. We also predicted 16,006 protein-coding genes from the generated assembly, and 99.35 (15,902 genes) of all protein-coding genes were annotated.

3. We found that the divergence time between *S. miscanthi* and its common ancestor shared with *Ac. pisum* was approximately 76.8-88.4 million years.

The assembly of this genome promotes research on the lifestyle and feeding specificity of aphids as well as their interactions with each other and other trophic levels and can serve as a resource for accelerating genome-assisted improvements in chemical drug resistant management as well as environmentally friendly aphid management.

Data availability

,The data supporting the results of this article has been deposited at DDBJ/ENA/GenBank under the accession SSSL00000000. The version described in this paper is version SSSL01000000.

Declarations

List of abbreviations

BUSCO: Benchmarking Universal Single-Copy Orthologs; CDS: Coding sequence; CLR: Continuous long reads; GO: Gene Ontology; KOG: EuKaryotic Orthologous Groups; KEGG: Kyoto Encyclopedia of Genes and Genomes; LINE: Long interspersed nuclear element; LTR: Long terminal repeat; NGS: Next-generation sequencing; NR: Nonredundant protein; NT: Nonredundant nucleotide; TrEMBL: Translation of European Molecular Biology Laboratory.

Author contributions

JF and JLC conceived the project; QL, QZ, YZ and YGQ collected aphid populations across China; XJ and QZ raised the aphids; JF and SYZ performed the observations on an ultra-depth three-dimensional microscope; XJ and YGQ collected the samples for both

genome and transcriptome sequencing; QZ, XJ and JF isolated the genomic DNA for both the 19-mer analysis and genome sequencing; JF and QZ isolated the total RNA for transcriptome sequencing; JF and HY performed the genome as well as transcriptome assembly, annotated the genome and conducted other data analysis; QL and YZ took the photographs of *S. miscanthi*, and JF and HY wrote the manuscript.

Ethics Statement

This statement is not required for experiments with *S. miscanthi*.

Acknowledgements

We thank Professor Chao Li from Xinjiang Agricultural University for drawing the Chinese map with origins of aphid populations. Thank Mr. Song Li and Huaigen Xin from Biomarker Technologies for the bioinformatics training.

Competing interests

The authors declare that they have no competing interests.

Funding

This research was sponsored by the National Key R & D Plan of China (nos. 2017YFD0200900, 2016YFD0300700 and 2017YFD0201700), the National Natural Science Foundation of China (nos. 31871966 and 31371946), the State Modern Agricultural Industry Technology System (CARS-22-G-18), and the China Scholarship Council (201703250048).

References

[1] Tao C. Aphid- fauna of China. Sci Year Book Taiwan Mus. 1961;4:35-44.

[2] Zhang G. Aphids in agriculture and forestry of northwest China. 1st ed. Beijing:

China Environmental Science; 1999.

[3] The International Aphid Genomics Consortium. Genome sequence of the pea aphid *Acyrthosiphon pisum*. PLoS Biol. 2010;8:e1000313.

[4] Mathers TC, Chen Y, Kaithakottil G, Legeai F, Mugford ST, Baa-Puyoulet P, et al. Rapid transcriptional plasticity of duplicated gene clusters enables a clonally reproducing aphid to colonise diverse plant species. Genome Biol. 2017;18:27.

[5] Wenger JA, Cassone BJ, Legeai F, Johnston JS, Bansal R, Yates AD, et al. Whole genome sequence of the soybean aphid, *Aphis glycines*. Insect Biochem Mol Biol. 2017. doi: 10.1016/j.ibmb.2017.01.005.

[6] Nicholson SJ, Nickerson ML, Dean M, Song Y, Hoyt PR, Rhee H, et al. The genome of *Diuraphis noxia*, a global aphid pest of small grains. BMC Genomics. 2015;16:429.

[7] Thorpe P, Escudero-Martinez CM, Cock PJA, Eves-van den Akker S, Bos JIB. Shared transcriptional control and disparate gain and loss of *Aphid parasitism* genes. Genome Biol Evol. 2018;10:2716-33.

[8] Quan Q, Hu X, Pan B, Zeng B, Wu N, Fang G, et al. Draft genome of the cotton *Aphid Aphis gossypii*. Insect Biochem Mol Biol. 2019;105:25-32.

[9] Chen W, Shakir S, Bigham M, Fei Z, Jander G. Genome sequence of the corn leaf aphid (*Rhopalosiphum maidis* Fitch). bioRxiv. 2018;8:1-12.

[10] Koren S, Walenz BP, Berlin K, Miller JR, Bergman NH, Phillippy AM. Canu: scalable and accurate long-read assembly via adaptive k-mer weighting and repeat separation. Genome Res. 2017;27:722-36.

[11] Chakraborty M, Baldwin-Brown JG, Long AD, Emerson JJ. Contiguous and accurate de novo assembly of metazoan genomes with modest long read coverage. Nucleic Acids Res. 2016;44:e147.

[12] Chin CS, Peluso P, Sedlazeck FJ, Nattestad M, Concepcion GT, Clum A, et al. Phased diploid genome assembly with single-molecule real-time sequencing. Nat Methods. 2016;13:1050-4.

[13] Simão FA, Waterhouse RM, Ioannidis P, Kriventseva EV, Zdobnov EM. BUSCO: assessing genome assembly and annotation completeness with single-copy orthologs. Bioinformatics. 2015;31:3210-2.

[14] Xie T, Zheng JF, Liu S, Peng C, Zhou YM, Yang QY, et al. De novo plant genome assembly based on chromatin interactions: a case study of *Arabidopsis thaliana*. Mol Plant. 2015;8:489-92.

[15] Parra G, Bradnam K, Korf I. CEGMA: a pipeline to accurately annotate core genes in eukaryotic genomes. Bioinformatics. 2007;23:1061-7.

[16] Xu Z, Wang H. LTR_FINDER: an efficient tool for the prediction of full-length LTR retrotransposons. Nucleic Acids Res. 2007;35:W265-8.

[17] Han Y, Wessler SR. MITE-Hunter: a program for discovering miniature inverted-repeat transposable elements from genomic sequences. Nucleic Acids Res. 2010;38:e199.

[18] Price AL, Jones NC, Pevzner PA. De novo identification of repeat families in large genomes. Bioinformatics. 2005;21:i351-8.

[19] Edgar RC, Myers EW. PILER: identification and classification of genomic repeats.

Bioinformatics. 2005;21:i152-8.

[20] Tarailo-Graovac M, Chen N. Using RepeatMasker to identify repetitive elements in genomic sequences. Curr Protoc Bioinformatics. 2009;Chapter 4:Unit 4.10.

[21] Jurka J, Kapitonov VV, Pavlicek A, Klonowski P, Kohany O, Walichiewicz J. Repbase update, a database of *Eukaryotic* repetitive elements. Cytogenet Genome Res. 2005;110:462-7.

[22] Wicker T, Sabot F, Hua-Van A, Bennetzen JL, Capy P, Chalhoub B, et al. A unified classification system for eukaryotic transposable elements. Nat Rev Genet. 2007;8:973-82.

[23] Stanke M, Waack S. Gene prediction with a hidden Markov model and a new intron submodel. Bioinformatics. 2003;19:ii215-25.

[24] Majoros WH, Pertea M, Salzberg SL. TigrScan and GlimmerHMM: two open source ab initio eukaryotic gene-finders. Bioinformatics. 2004;20:2878-9.

[25] Korf I. Gene finding in novel genomes. BMC Bioinformatics. 2004;5:59.

[26] Blanco E, Parra G, Guigo R. Using geneid to identify genes. Curr Protoc Bioinformatics. 2007;Chapter 4:Unit 4.3.

[27] Burge C, Karlin S. Prediction of complete gene structures in human genomic DNA. J Mol Biol. 1997;268:78-94.

[28] Keilwagen J, Wenk M, Erickson JL, Schattat MH, Grau J, Hartung F. Using intron position conservation for homology-based gene prediction. Nucleic Acids Res. 2016;44:e89.

[29] Campbell MA, Haas BJ, Hamilton JP, Mount SM, Buell CR. Comprehensive analysis

of alternative splicing in rice and comparative analyses with *Arabidopsis*. BMC Genomics. 2006;7:327.

[30] Haas BJ, Salzberg SL, Zhu W, Pertea M, Allen JE, Orvis J, et al. Automated eukaryotic gene structure annotation using EVidenceModeler and the program to assemble spliced alignments. Genome Biol. 2008;9:R7.

[31] Altschul SF, Gish W, Miller W, Myers EW, Lipman DJ. Basic local alignment search tool. J Mol Biol. 1990;215:403-10.

[32] Marchler-Bauer A, Lu S, Anderson JB, Chitsaz F, Derbyshire MK, DeWeese-Scott C, et al. CDD: a conserved domain database for the functional annotation of proteins. Nucleic Acids Res. 2011;39:D225-9.

[33] Koonin EV, Fedorova ND, Jackson JD, Jacobs AR, Krylov DM, Makarova KS, et al. A comprehensive evolutionary classification of proteins encoded in complete eukaryotic genomes. Genome Biol. 2004;5:R7.

[34] Dimmer EC, Huntley RP, Alam-Faruque Y, Sawford T, O'Donovan C, Martin MJ, et al. The UniProt-GO annotation database in 2011. Nucleic Acids Res. 2012;40:D565-70.

[35] Kanehisa M, Goto S. KEGG: Kyoto encyclopedia of genes and genomes. Nucleic Acids Res. 2000;28:27-30.

[36] Boeckmann B, Bairoch A, Apweiler R, Blatter M-C, Estreicher A, Gasteiger E, et al. Phan I: the SWISS-PROT protein knowledgebase and its supplement TrEMBL in 2003. Nucleic Acids Res. 2003;31:365-70.

[37] Li L, Stoeckert CJ, Roos DS. OrthoMCL: identification of ortholog groups for

eukaryotic genomes. Genome Res. 2003;13:2178-89.
[38] Guindon S, Dufayard JF, Lefort V, Anisimova M, Hordijk W, Gascuel O. New algorithms and methods to estimate maximum-likelihood phylogenies: assessing the performance of PhyML 3.0. Syst Biol. 2010;59:307-21.
[39] Yang Z, Rannala B. Bayesian estimation of species divergence times under a molecular clock using multiple fossil calibrations with soft bounds. Mol Biol Evol. 2006;23:212-26.
[40] Hedges SB, Marin J, Suleski M, Paymer M, Kumar S. Tree of life reveals clock-like speciation and diversification. Mol Biol Evol. 2015;32:835-45.

Figure legends

Figure 1. Winged and wingless *S. miscanthi*. a. Winged adult. b. Wingless adult.

Figure 2. The most distinct morphological structures of *S. miscanthi* compared with *S. avenae*. Wingless/Winged a: the 3rd segment of the antennae of a wingless/winged adult; Wingless/Winged b: the lengths of both the cornicle and caudae of a wingless/winged adult; Wingless/Wingless c: the hair inserted into the caudae of a wingless/winged adult.

Figure 3. 19-mer distribution for genome size prediction of *S. miscanthi.*

Figure 4. Hi-C contact heatmap of the *S. miscanthi* genome.

Figure 5. The phylogenetic relationships of *S. miscanthi* with other arthropods.

Tables

Table 1. Assessment results based on two strategies.

Genome feature/assessment strategy	19-mer analysis	PacBio
Genome size (Mb)	393.12	397.90
GC content (%)	31.70	30.25
Repeat sequence content (%)	35.07	24.14
Heterozygosity (%)	0.98	0.57

Table 2. Assembly statistics of *S. miscanthi* genome and 7 other aphid genomes based mainly on NGS.

Genome assembly/species	*S. miscanthi*	*R. padi*	*D. noxia*	*Ac. pisum*	*Ap. glycines*	*M. persicae*	*M. cerasi*	*Ap. gossypii*
Assembly size (Mb)	397.9	319.4	393.0	541.6	302.9	347.3	405.7	294.0
Contig count	1,148	16,689	49,357	60,623	66,000	8,249	56,508	22,569
Contig N50 (bp)	1,638,329	96,831	12,578	28,192	15,844	71,400	17,908	45,572

Scaffold count	656	15,587	5,641	23,924	8,397	4,018	49,286	4,724
Scaffold N50 (bp)	36,263,045	116,185	397,774	518,546	174,505	435,781	23,273	437,960
Genome annotation								
Gene count	16,006	26,286	19,097	36,195	17,558	18,529	28,688	14,694
Mean gene length (kb)	7.805	1,543	1.316	1.964	1.520	1.839	1,222	1.964
Mean exon count per gene	6.7	5.20	3.0	5.0	6.2	6.1	3.7	10.1
Mean exon length (bp)	288	162	249.0	394.7/429	246	299	178	218

Table 3. Summary of *S. miscanthi* genome assembly.

Statistics	Draft scaffolds	Corrected by HI-C
Contig number	1,039	1,167
Contig length	397,907,165	397,907,165
Contig N50 (bp)	2,049,770	1,565,814
Contig N90 (bp)	256,083	185,510
Contig max (bp)	11,219,273	10,100,000
Gap number/gap total length (bp)	0	0

Table 4. Detailed classification of repeats in the *S. miscanthi* genome assembly.

Type	Number	Length (bp)	Rate (%)
Class I	194093	51169345	12.86
DIRS	1,289	695,762	0.17
LINE	40,230	10,832,765	2.72
LTR/Copia	2,438	742,051	0.19
LTR/Gypsy	18,807	6,949,790	1.75
LTR/Unknown	7,534	3,195,404	0.8

PLE|LARD	115,765	28,920,417	7.27
SINE	6,665	1,075,456	0.27
SINE|TRIM	15	5,478	0
TRIM	1,116	1,281,655	0.32
Class I Unknown	234	26,384	0.01
Class II	188,820	44,184,063	11.1
Crypton	299	20,282	0.01
Helitron	5,688	1,871,785	0.47
MITE	7,972	1,434,924	0.36
Maverick	7,888	3,289,168	0.83
TIR	89,268	22,913,523	5.76
Class II Unknown	77,705	15,793,696	3.97
Potential Host Gene	926	251,812	0.06
SSR	2,611	381,142	0.1
Unknown	74,204	18,832,522	4.73
Identified	386,450	105,110,753	26.42
Total	460,654	123,943,275	31.15

Figure1

Click here to download Figure Figure 1.tiff

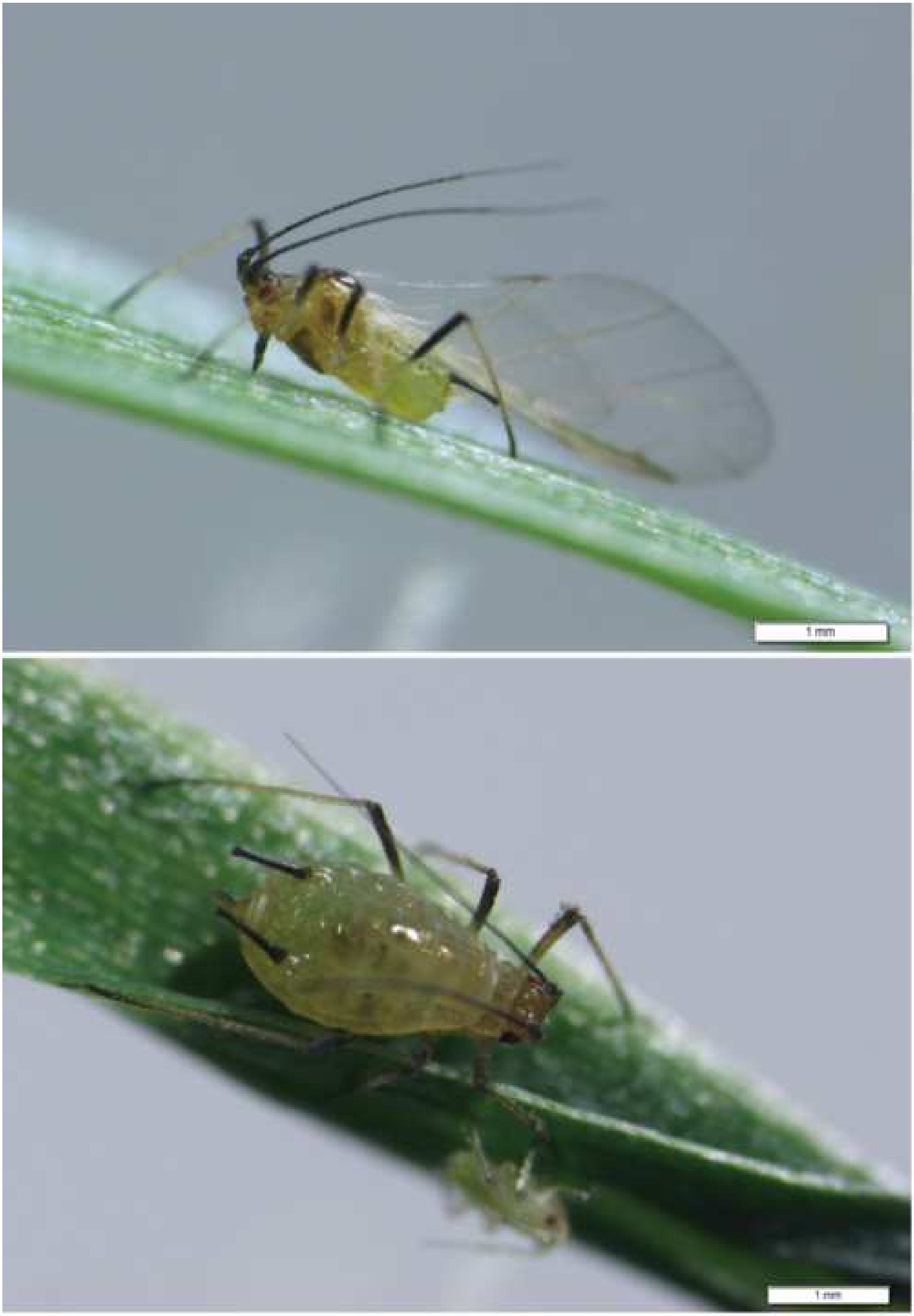

Figure2

Click here to download Figure figure2_3.jpg

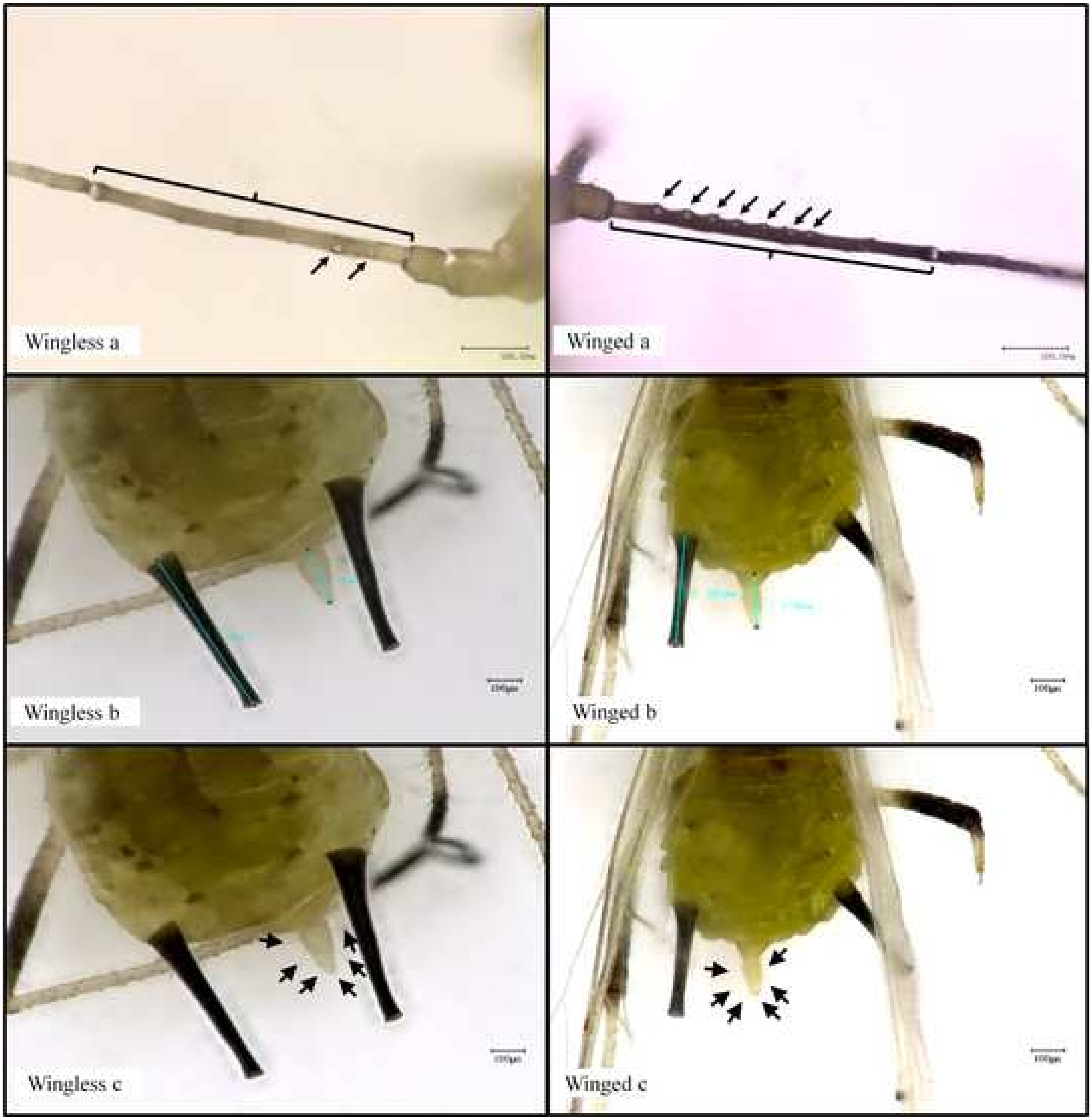

 Click here to download Figure Figure3_kmeranalysis.png

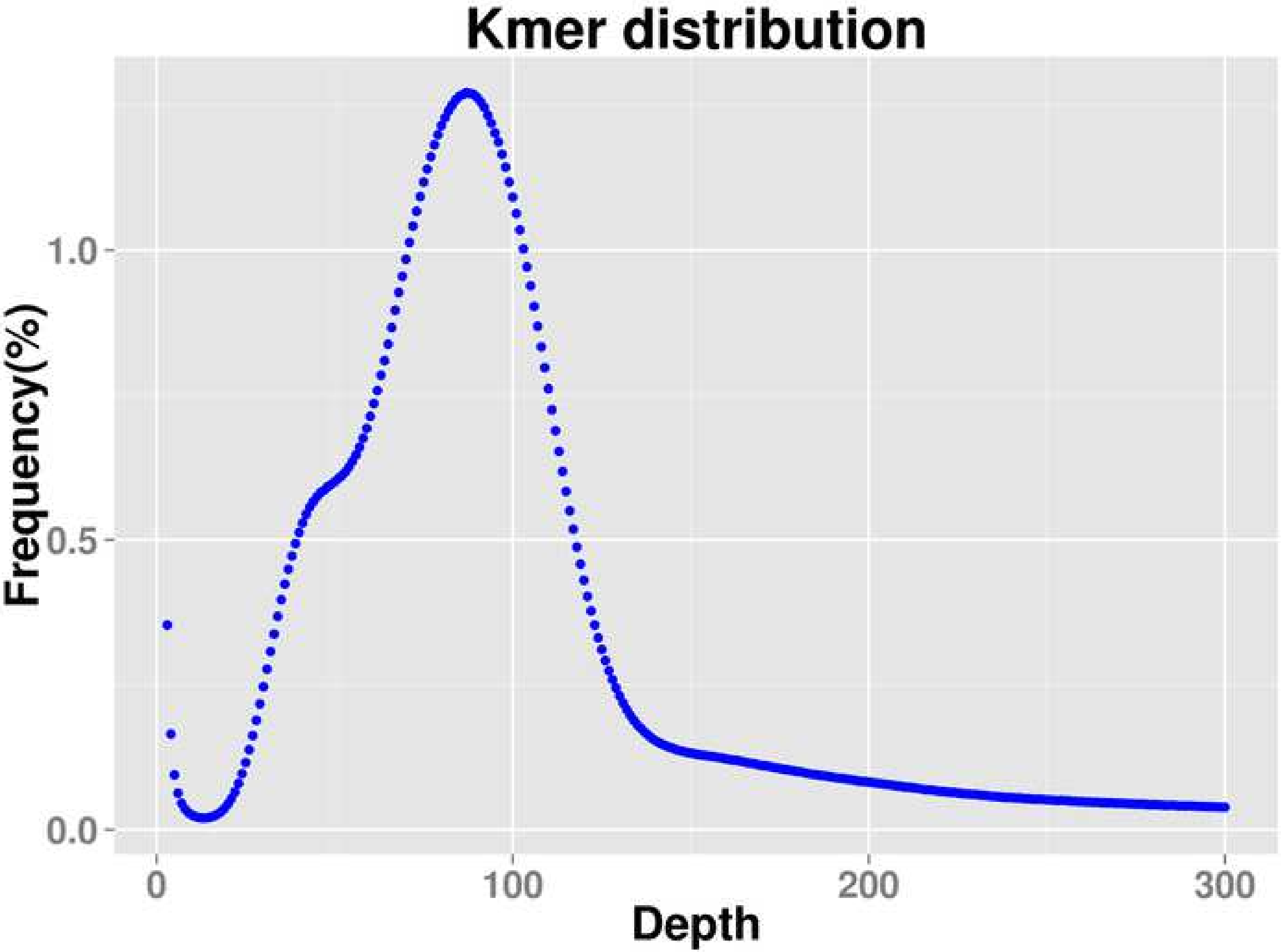

Figure4

Click here to download Figure Figure 4 HICheatmap.jpeg

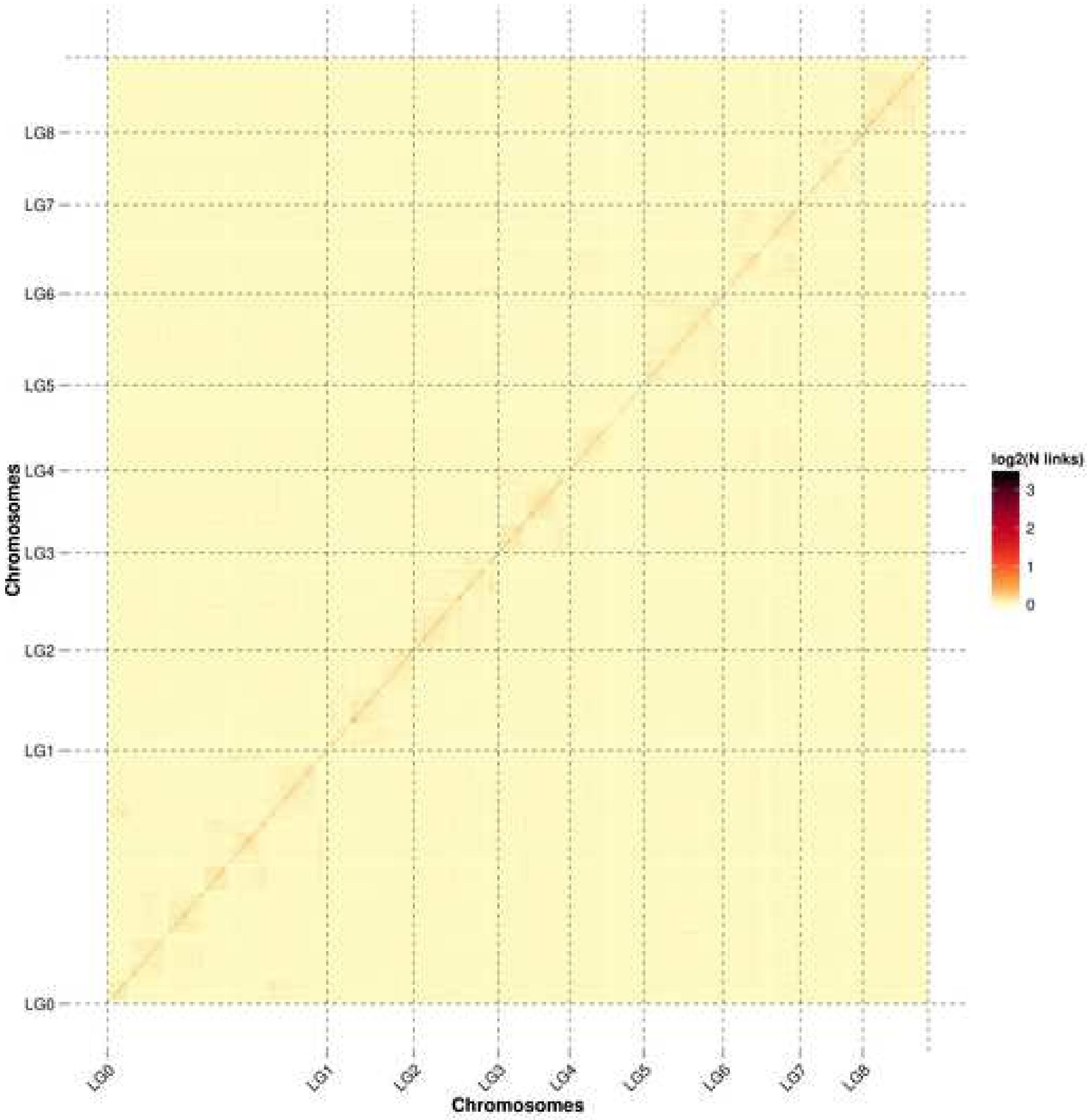

Figure5

Click here to download Figure Figure5_phylogenetic tree.jpg

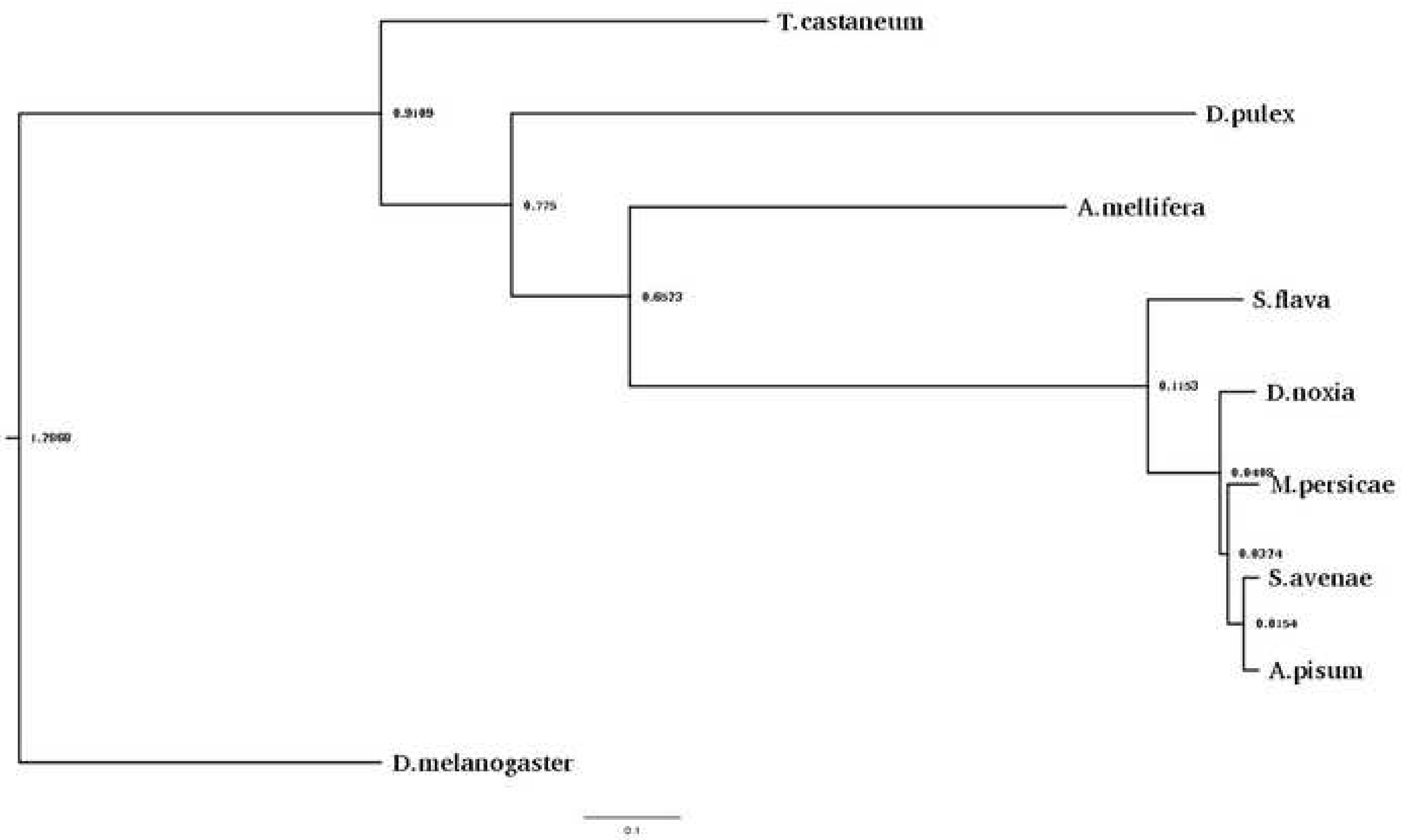

Click here to access/download
Supplementary Material
supplementary materials.docx
