# Supplementary material for: A chromosome-level draft genome of the grain aphid Sitobion miscanthi
Source: Gigascience. 2019 Aug 20;8(8):giz101. doi: 10.1093/gigascience/giz101 (PMC6701489; doi:10.1093/gigascience/giz101)
Supplement: giz101_GIGA-D-19-00137_Revision_1 [file giz101_giga-d-19-00137_revision_1.pdf]

GigaScience

A chromosome-level draft genome of the grain aphid Sitobion miscanthi

--Manuscript Draft--

**Manuscript Number:**	GIGA-D-19-00137R1
**Full Title:**	A chromosome-level draft genome of the grain aphid Sitobion miscanthi
**Article Type:**	Data Note
**Funding Information:**	National Key R & D Plan of China (2017YFD0200900) — Dr. Jia Fan<br>National Natural Science Foundation of China (31871966) — Dr. Jia Fan<br>State Modern Agricultural Industry Technology System (CARS-22-G-18) — Dr. Jia Fan<br>China Scholarship Council (201703250048) — Dr. Jia Fan<br>National Key R & D Plan of China (2016YFD0300700) — Prof. Julian Chen<br>National Key R & D Plan of China (2017YFD0201700) — Prof. Julian Chen<br>National Natural Science Foundation of China (31371946) — Prof. Julian Chen

Funder	Recipient
National Key R & D Plan of China (2017YFD0200900)	Dr. Jia Fan
National Natural Science Foundation of China (31871966)	Dr. Jia Fan
State Modern Agricultural Industry Technology System (CARS-22-G-18)	Dr. Jia Fan
China Scholarship Council (201703250048)	Dr. Jia Fan
National Key R & D Plan of China (2016YFD0300700)	Prof. Julian Chen
National Key R & D Plan of China (2017YFD0201700)	Prof. Julian Chen
National Natural Science Foundation of China (31371946)	Prof. Julian Chen

Abstract:

Background: Sitobion miscanthi is an ideal model for studying host plant specificity, parthenogenesis-based phenotypic plasticity, and interactions between insects and other species of various trophic levels, such as viruses, bacteria, plants and natural enemies. However, the genome information for this species has not been published yet. Here, we analyzed the entire genome of a female aphid colony using long-read sequencing and Hi-C data to generate chromosome-length scaffolds and a highly contiguous genome assembly.

Results:

1.The final draft genome assembly from 33.88 Gb of raw data was approximately 397.90 Mb with a 2.05 Mb contig N50. Nine chromosomes were further assembled based on Hi-C data to a 377.19 Mb final size with a 36.26 Mb scaffold N50.

2.The identified repeat sequences accounted for 26.41% of the genome, and 16,006 protein-coding genes were annotated. According to the phylogenetic analysis, S. miscanthi is closely related to Acyrthosiphon pisum, with S. miscanthi diverging from their common ancestor approximately 25.0-44.9 million years ago.

Conclusions: We generated a high-quality draft of the Sitobion miscanthi genome. This genome assembly promotes research on the lifestyle and feeding specificity of aphids and their interactions with each other and species at other trophic levels. It can serve as a resource for accelerating genome-assisted improvements in chemical drug resistant management and environmentally friendly aphid management.

**Corresponding Author:**	Jia Fan<br><br>CHINA
**Corresponding Author Secondary Information:**	
**Corresponding Author's Institution:**	
**Corresponding Author's Secondary Institution:**	
**First Author:**	Xin Jiang
**First Author Secondary Information:**	
**Order of Authors:**	Xin Jiang<br>Qian Zhang

Powered by Editorial Manager® and ProduXion Manager® from Aries Systems Corporation

	Yaoguo Qin
	Hang Yin
	Siyu Zhang
	Qian Li
	Yong Zhang
	Jia Fan
	Julian Chen
**Order of Authors Secondary Information:**	

Response to Reviewers:

Dear editor and reviewers,

Thank you for your concerning on our manuscript entitled “A chromosome-level draft genome of the grain aphid Sitobion miscanthi” (Manuscript ID: GIGA-D-19-00137). We appreciate very much for your helpful comments and constructive suggestions on this manuscript. Those comments are all very helpful for us to improve our manuscript. Please receive the revised manuscript. All the co-authors and I have carefully checked the manuscript and revised it according to your suggestions and the comments. Please let us know if you have any question or need any further information about our manuscript.
Sincerely yours,

Jia Fan
Dr. Jia Fan
State Key Laboratory for Biology of Plant Diseases and Insect Pests
Institute of Plant Protection
Chinese Academy of Agricultural Sciences
Beijing 100193, China
Email: jfan@ippcaas.cn

Reviewer 1:
The authors describe the genome of S. miscanthi using modern HiSeq methods. This manuscript and these data will add to the rapidly growing number of important aphid pest species with complete genome sequences. While the data appears solid, and the genome is high quality, I have some major comments on the manuscript:
Major:
1. The manuscript seems to weave two tales, but only one (the genome) is strong enough for publication. The authors try to incorporate what appears to be a sibling species issue, with very light discussion and detail on apparent mis-identification or confusion between S. miscanthi and S. avenae. They tried to add some survey data (with collections in 17 different Chinese locations), but this section does not include any data as far as sampling, variation, etc. While the troubling taxonomy may be an issue for this system the authors need to make a decision to either remove this section and focus on the genome, or be ready to add more detail to the manuscript to support their claim of S. miscanthi. To keep the manuscript simple, I suggest that the authors follow the former—the inclusion of the population data is not necessary, in my opinion, for publication.
Response: According to the reviewer’s suggestions, we have removed the species identification section to make the manuscript simple and focus on the genome.
2. In many bioinformatic analyses, the authors seem to not have a workflow, and rather just use any sequencing software that may fit their data. While it is ok to use multiple programs, using them without a rationale makes it seem like the authors are grasping at straws. Furthermore, there are no details for each program? Why did they use all the programs? What did they expect to see and why did they not see it initially and then had to use a different program? How do they know they got an adequate answer with one program and not the other? For example, the section starting on L148 contains multiple programs without any output, rationale or discussion on the different programs.
Response:We have added more detail in the sections on “genome assembly” as well as “annotation” in our manuscript (Line 87-108):
De novo genome assembly with long reads was performed using two pipelines, Canu

and wtdbg. Because of the high heterozygosity of Sitobion miscanthi, in the correction step, Canu first selects longer seed reads with the settings ‘genomeSize=400000000’ and ‘corOutCoverage=50’, then detects overlapping raw reads through the highly sensitive overlapper MHAP (mhap-2.1.2, option ‘corMhapSensitivity=low/normal/high’), and finally performs error correction with the falcon_sense method (option ‘correctedErrorRate=0.025’). In the next step, with the default parameters, error-corrected reads are trimmed to remove unsupported bases and hairpin adapters to obtain the longest supported range. In the last step, Canu generates the draft assembly using the longest 80 coverage-trimmed reads with Canu v1.5 [13] to output more corrected reads and be more conservative at picking the error rate for the assembly to try to maintain haplotype separation.
Wtdbg is an SMS data assembler that constructs fuzzy Brujin graphs (available at https://github.com/ruanjue/wtdbg). Wtdbg first generates a draft assembly with the command ‘wtdbg -i pbreads.fasta -t 64 -H -k 21 -S 1.02 -e 3 -o wtdbg’. The use of error-corrected reads from Canu results in better assembly performance. Then, a consensus assembly is obtained with the command ‘wtdbg-cns -t 64 -i wtdbg.ctg.lay -o wtdbg.ctg.lay.fa -k 15’.
To improve genome contiguity, two assemblies generated from the Canu and wtdbg pipelines were merged with three rounds of quickmerge [14]. Quickmerge uses contigs from wtdbg as query input and contigs from Canu as ref input. The two contigs are aligned through mummer (v4.0.0, available at https://github.com/mummer4/mummer), with the nucmer parameters ‘-b 500 -c 100 -l 200 -t 12’ and delta-filter parameters ‘-i 90 -r -q’, and then merged through quickmerge with the parameters ‘-hco 5.0 -c 1.5 -l 100000 -ml 5000’.

Minor Comments
3. l21: is a worldwide, sap-sucking pest of wheat; l24: "This species was..." this sentence is probably not relevant to include in the abstract. Instead replace the sentence with one that explains why the genome would be so important.
Response: We revised this as follows (from Line 21 to Line 24):
Sitobion miscanthi is an ideal model for studying host plant specificity, parthenogenesis-based phenotypic plasticity, and interactions between insects and other species of various trophic levels, such as viruses, bacteria, plants and natural enemies. However, the genome information for this species has not been published yet.
4. l40: This sentence needs to be broken up, it is far too long and contains several different ideas
Response: This sentence has been broken up as follows (Line 35-39):
We generated a high-quality draft of the Sitobion miscanthi genome. This genome assembly promotes research on the lifestyle and feeding specificity of aphids and their interactions with each other and species at other trophic levels. It can serve as a resource for accelerating genome-assisted improvements in chemical drug resistant management and environmentally friendly aphid management.
5. l53: remove product
l54: replace scope with range, and replace the simple with its simple
l55: ideal model is redundant
l62: remove are available. Furthermore, I don't think you need to list where these genomes are since each aphid genome is properly cited
l63: italicize A .gossypii
Response: We thank for the reviewer’s very meticulous review. According to the reviewer's comments, they were revised as follows:
In line 51 of revised manuscript, product was removed.
Scope was replaced with range in line 48, and the simple was replaced with its simple in line 48-49.
In line 50, ideal model was changed into significant.
Fully consider the reviewer’s helpful advice, this sentence (Line 52-56) was re-organized: Genomes with annotation information from a total of 8 aphid species, namely the pea aphid Acyrthosiphon pisum [2], peach aphid Myzus persicae [3], soybean aphid Aphis glycines [4], Russian wheat aphid Diuraphis noxia [5], cherry-oat aphid Rhopalosiphum padi [6], and black cherry aphid Myzus cerasi [6], the cotton aphid Aphis gossypii [7], and the corn leaf aphid Rhopalosiphum maidis [8] are available.
In line 59, Aphis gossypii is italicize now.
6. L79: How do the authors know that nymphs collected within 12 h did not feed?

Powered by Editorial Manager® and ProduXion Manager® from Aries Systems Corporation

Response: Mother aphids were placed into culture dishes (dimeter of 9 cm) with moist absorbent paper on the bottom for 12 h. No newborn nymphs were fed during this period. Newborn nymphs within 12 h without feeding were collected for genome sequencing. We have added this sentence into the manuscript to clarify(Line 67-70). Thank you very much.

7. Figure 2: What am I looking at here? The figures have labels but the caption needs more detail to explain to the reader what is relevant

Response: According to the reviewer's first comment, the species identification section was removed from this manuscript.

8. L93: This section needs a better citation for the methods other than including a protocol

Response: According to the reviewer's comment, two citations cited as [11] and [12] have been inserted into the manuscript (Line 77).

11. Altschul SF, Gish W, Miller W, Myers EW, Lipman DJ. Basic local alignment search tool. Journal of Molecular Biology. 1990;215:403-10.

12. Li R, Li Y, Kristiansen K, Wang J. SOAP: short oligonucleotide alignment program. Bioinformatics. 2008;24:713-14.

9. L99: remove finally

Response: We removed "finally" (Line 78).

10. L129: I think more detail is needed here for Hi-C sequencing other than just citing 2 different papers

Response: According to the reviewer's suggestion, we have detailed the Hi-C sequencing in line 127-149 as follows,

Hi-C fragment libraries were constructed with insert sizes of 300-700 bp and sequenced on the Illumina platform. Adapter sequences of raw reads were trimmed, and low-quality PE reads were removed for clean data. The clean Hi-C reads were first truncated at the putative Hi-C junctions, and then the resulting trimmed reads were aligned to the assembly results with BWA software [17]. Only uniquely alignable pairs reads whose mapping quality was more than 20 remained for further analysis. Invalid read pairs, including Dangling-End and Self-cycle, Re-ligation and Dumped products, were filtered by HiC-Pro (v2.8.1) [18].

In total, 38.44% of unique mapped read pairs were valid interaction pairs for scaffold correction and were used to cluster, order and orient scaffolds onto chromosomes by LACHESIS.

Before chromosome assembly, we first performed a preassembly for the error correction of scaffolds, which required the splitting of scaffolds into segments of 50 kb on average. The Hi-C data were mapped to these segments using BWA (version 0.7.10-r789) software. The uniquely mapped data were retained to perform assembly by using LACHESIS software. Any two segments that showed inconsistent connections with information from the raw scaffold were checked manually. These corrected scaffolds were then assembled with LACHESIS. Parameters for running LACHESIS included CLUSTER_MIN_RE_SITES, 70; CLUSTER_MAX_LINK_DENSITY, 1; ORDER_MIN_N_RES_IN_TRUN, 19; ORDER_MIN_N_RES_IN_SHREDS, 19. After this step, placement and orientation errors exhibiting obvious discrete chromatin interaction patterns were manually adjusted. Finally, 774 scaffolds (representing 97.48% of the total length) were anchored to 9 chromosomes (Figure 3, Table S1).

11. L132: BWA software needs a citation

Response: According to the reviewer's comment, one citation of [17] has been inserted into the manuscript (Line 131).

17. Li H, Richard D. Fast and accurate short read alignment with Burrows–Wheeler transform. Bioinformatics. 2009;25(14):1754-60.

12. Figure 4 is unreadable.

Response: We are very sorry for this mistake. Figure 4 has been uploaded again, and it is also shown below.

13. L140: do they have a karyotype citation?

Response: Yes, two citations of [9] and [10] have been inserted into the manuscript (Line 61).

9. Kuznesova VG, Shaposhnikoy GKH. The chromosome numbers of the aphids (Homoptera, Aphidinea) of the world fauna. Entomological Review. 1973;52:78-96.

10. Chen XD, Zhang GX. The chromosome numbers of the aphids in Beijing region.Acta Zoologica Sinica. 1985;31(1):12-9.

14. L147: Similar to the assembly, why all these programs? And what data did all these programs give (and where is it)? What is the workflow for this part of the study?

Powered by Editorial Manager® and ProduXion Manager® from Aries Systems Corporation

Response: We have added more details for this part from line 155 to line 164.
To identify tandem repeats, we utilized 4 software programs, namely LTR_FINDER (v1.0.5) [19], MITE-Hunter (v1.0.0) [20], RepeatScout (v1.0.5) [21], and PILER-DF (v1.0) [22], to build a de novo repeat library based on our assembly with the default settings. Subsequently, the predicted repeats were classified using PASTEClassifier (v1.0) [23] and merged with Repbase (19.06) [24]. Finally, using the resulting repeat database as the final repeat library, RepeatMasker v4.0.5 (RepeatMasker, RRID: SCR 012954) [25] was used to identify repetitive sequences in the A. nanus genome with the following parameters: "-nolow -no is -norna -engine wublast." The repeat sequences accounted for 31.15% of the S. miscanthi genome, including identified repeat sequences (26.42% of the genome), based on the de novo repeat library (Table 4).
15. L165: How were the transcript assembled? And how good is this assembly?
Response: We have detailed the method of transcript assembled and quality assessed in line 174-176. Transcripts were assembled based on the genome-guide trinity.
The quality of the transcripts was assessed by the proportion of gene regions covered by these transcripts, the higher being better. In this case, the proportion was 85.66%.
16. L235: remove the ,
Response: According to the reviewer's comments, we have removed "the" (Line 241).
17. Figure 5 is poor quality, I cannot read the node support. Plus, S. avenae is included--I assume this is miscanthi?
Response: We are very sorry for the poor quality of Figure 5, and the current Figure 5 is updated as follows:

Reviewer 2:
The authors sequenced the genome of Sitobion miscanthi using long-read sequencing and Hi-C. They generated chromosome-length scaffolds and assembled the collected data to produce nine chromosomes to a final size of 377.19 Mb. A total of 16,006 PCGs were annotated and found that 26.41% of the genome consists of repetitive sequences. The manuscript presents a good genome draft of a Hemipteran species, previously not reported and as such merit publication. However, the paper contains many discrepancies that require addressing.
1. Also the grammar should be improved.
Responses: According to the reviewer's suggestion, to improve our manuscript, the revised version was edited by a professional language editor before submission.
2. The legend of fig 2 should be improved as it is unclear which samples represent S. avenae and S. marcanthi.
Response:The species identification section, including Figure 2, was removed to simplify this manuscript and focus on the genome.
3. The phylogenetic analysis (Fig 5) provides insight into the relationships of different arthropod species relative to S. miscanthi, but S. miscanthi doesn't feature in the figure? So, I fail to see what the value of this figure is. The resolution of the figure should also be improved as it is impossible to read the values on the branches.
Response: We are very sorry for this, and the current Figure 5 was updated and uploaded in the submission system.
4. Table 1 give the statistics of all the sequenced aphid genomes, but doesn't include the most recent/updated versions of the different genomes (e.g. D. noxia actually has more genes, see the paper in Standards in Genomic Sciences 2018, https://doi.org/10.1186/s40793-017-0307-6). Please update to represent the most recent data.
Response: The most recent data have been updated in Table 2.
5. It is interesting that the genome size estimated on k-mer analysis differs so much from the final genome estimation, please explain/discuss.
Response: The genome size assessed by the survey map is 393.12 Mb, and the final genome size is 397.95 Mb. The overall rate of heterozygosity (~0.98) indicated that this genome may have a high rate of heterozygous regions, which should be carefully considered in subsequent assembly processes. A Canu assembly of 585.29 Mb and 6,014 contigs were generated, with most haplotypes associated with diploid populations. High heterozygosity of the genome is the main cause of a large genome assembly.
6. Sipha flava is not listed as a genome with sequence information - so where were the data obtained from to conduct the homology-based predictions? Please clarify.
Response: This species, Sipha flava, cited in our manuscript is the only aphid whose genome has not been formally published. The website where its sequence information

Powered by Editorial Manager® and ProduXion Manager® from Aries Systems Corporation

	can be downloaded is shown in our manuscript (Lines 206-207) ftp://ftp.ncbi.nlm.nih.gov/genomes/all/GCF/003/268/045/GCF_003268045.1_YSA_version1 196 /GCF_003268045.1_YSA_version1_genomic.fna.gz)<br><br>Minor issues:<br>7. Lines 59-64, and again line 145: "information of 6 aphid species"' - but I count 8?<br><br>Response: According to the reviewer's comments, we have carefully corrected them all (Line 52, 152).<br>8. Line 188: Refers to functional annotation of PCGs but cite Fig. 3 (k-mer analysis)?<br>Response: Yes, it should be Figure S3, we have corrected it in the manuscript (Line 199). We invited a language editor to minimize similar editorial issues.<br>9. There are many minor editorial issues that needs addressing (e.g., lack of commas, lines 59-64; in appropriate comma use, line 235; not using the demonstrative pronoun "the", line 73 etc.,)<br>Response: We invited a language editor to minimize similar editorial issues. Thank you very much.
Additional Information:	
Question	Response
Are you submitting this manuscript to a special series or article collection?	No
**Experimental design and statistics**<br><br>Full details of the experimental design and statistical methods used should be given in the Methods section, as detailed in our Minimum Standards Reporting Checklist. Information essential to interpreting the data presented should be made available in the figure legends.<br><br>Have you included all the information requested in your manuscript?	Yes
**Resources**<br><br>A description of all resources used, including antibodies, cell lines, animals and software tools, with enough information to allow them to be uniquely identified, should be included in the Methods section. Authors are strongly encouraged to cite Research Resource Identifiers (RRIDs) for antibodies, model organisms and tools, where possible.<br><br>Have you included the information requested as detailed in our Minimum Standards Reporting Checklist?	Yes

**Availability of data and materials**<br><br>All datasets and code on which the conclusions of the paper rely must be either included in your submission or deposited in publicly available repositories (where available and ethically appropriate), referencing such data using a unique identifier in the references and in the "Availability of Data and Materials" section of your manuscript.<br><br>Have you have met the above requirement as detailed in our Minimum Standards Reporting Checklist?	Yes

Manuscript

Click here to view linked References

Click here to access/download;Manuscript;Genome of Sitiobion miscanthi revision 1 unTRACKed0624.doc

A chromosome-level draft genome of the grain aphid *Sitobion miscanthi*

Xin Jiang*, Qian Zhang*, Yaoguo Qin*, Hang Yin, Siyu Zhang, Qian Li, Yong Zhang, Jia Fan†, Julian Chen†

Affiliations: All authors, State Key Laboratory for Biology of Plant Diseases and Insect Pests, Institute of Plant Protection, Chinese Academy of Agricultural Sciences, Beijing 100193, People's Republic of China. Jia Fan, Tel: +86-01062815934, E-mail: jfan@ippcaas.cn; Julian Chen, 86-10-62813685, E-mail: chenjulian@caas.cn; Xin Jiang, Tel: +86-01062815934, E-mail: 18911895763@163.com; Qian Zhang, Tel: +86-01062815934, E-mail: zhangqianelaine@163.com; Yaoguo Qin, Tel: +86-01062815934, E-mail: qinyg1018@163.com; Hang Yin, Tel: +86-01062815934, E-mail: yhang01@163.com; Siyu Zhang, Tel: +86-01062815934, E-mail: Zhangsiyu1567@163.com; Qian Li, Tel: +86-01062815934, E-mail: liqian0927@yeah.net; Yong Zhang, Tel: +86-01062815934, E-mail: zhangyongnky@163.com

†**Corresponding authors:** Jia Fan, State Key Laboratory for Biology of Plant Diseases and Insect Pests, Institute of Plant Protection, Chinese Academy of Agricultural Sciences, 2 Yuanmingyuan West Road, Haidian District, Beijing, 100193, P. R., China, 86-10-62815934, jfan@ippcaas.cn.

Julian Chen, State Key Laboratory for Biology of Plant Diseases and Insect Pests, Institute of Plant Protection, Chinese Academy of Agricultural Sciences, 2 Yuanmingyuan West Road, Haidian District, Beijing 100193, P. R., China, 86-10-62813685, chenjulian@caas.cn.

*Equal contribution

Abstract

***Background*:** *Sitobion miscanthi* is an ideal model for studying host plant specificity, parthenogenesis-based phenotypic plasticity, and interactions between insects and other species of various trophic levels, such as viruses, bacteria, plants and natural enemies. However, the genome information for this species has not been published yet. Here, we analyzed the entire genome of a female aphid colony using long-read sequencing and Hi-C data to generate chromosome-length scaffolds and a highly contiguous genome assembly.

***Results*:**

1. The final draft genome assembly from 33.88 Gb of raw data was approximately 397.90 Mb with a 2.05 Mb contig N50. Nine chromosomes were further assembled based on Hi-C data to a 377.19 Mb final size with a 36.26 Mb scaffold N50.
2. The identified repeat sequences accounted for 26.41% of the genome, and 16,006 protein-coding genes were annotated. According to the phylogenetic analysis, *S. miscanthi* is closely related to *Acyrthosiphon pisum*, with *S. miscanthi* diverging from their common ancestor approximately 25.0-44.9 million years ago.

***Conclusions*:** We generated a high-quality draft of the *Sitobion miscanthi* genome. This genome assembly promotes research on the lifestyle and feeding specificity of aphids and their interactions with each other and species at other trophic levels. It can serve as a resource for accelerating genome-assisted improvements in chemical drug resistant management and environmentally friendly aphid management.

Keywords: aphid, *Sitobion miscanthi*, *Sitobion avenae*, annotation, genome, long-read sequencing, Hi-C assembly

Data Description

Background

The grain aphid *Sitobion miscanthi* (Figure 1), a cereal specialist, is a globally distributed

sap-sucking pest of wheat and a dominant species in wheat-growing regions across China. It threatens wheat production in various ways such as pillaging nutrition from the host, transmitting pathogenic plant viruses, and defecating sticky honeydew that further obstructs photosynthesis and reduces wheat quality. Together with its highly specialized host range, its simple parasitic life cycle, pleomorphism, and alternation of complete and incomplete life cycles make *S. miscanthi* significant for both basic and applied research. This species was misidentified as *Sitobion avenae* in China [1]. Therefore, we sought to publish the genome information for *S. miscanthi* here. Genomes with annotation information from a total of 8 aphid species, namely the pea aphid *Acyrthosiphon pisum* [2], peach aphid *Myzus persicae* [3], soybean aphid *Aphis glycines* [4], Russian wheat aphid *Diuraphis noxia* [5], cherry-oat aphid *Rhopalosiphum padi* [6], and black cherry aphid *Myzus cerasi* [6], the cotton aphid *Aphis gossypii* [7], and the corn leaf aphid *Rhopalosiphum maidis* [8] are available. However, no genome information for *S. miscanthi* has been published. Here, we report the chromosome-level genome sequence of the *S. miscanthi* isolate Langfang-1, which exhibits higher-quality assembly data indexes than other scaffold-level aphid genomes. Most of the sequences assembled into 9 scaffolds, which supported a 2n=18 karyotype for *S. miscanthi* [9,10]. The repeat sequences and phylogenetic relationship of *S. miscanthi* with other insects were further analyzed.

Sampling

Langfang-1, a grain aphid (*S. miscanthi*) isolate that was originally collected from wheat in Hebei province, was kept in our laboratory for genome sequencing.

An isogenic colony was started from a single parthenogenetic female of *S. miscanthi* and was maintained on wheat (*Triticum aestivum*). Mother aphids were placed into culture dishes (dimeter of 9 cm) with moist absorbent paper on the bottom for 12 h. No newborn nymphs were fed during this period. Newborn nymphs within 12 h without feeding were collected for

genome sequencing. In addition, 100 aphids of 1st and 2nd instars and 50 winged and wingless aphids at the 3rd instar, 4th instar and adult stages were collected for transcriptome sequencing.

Genome size estimation

High-quality genomic DNA for sequencing using the Illumina platform (Illumina Inc., San Diego, CA, USA) and PacBio Sequel sequencing (Pacific Biosciences of California, Menlo Park, CA, USA) was extracted from the newborn nymphs mentioned above. The whole-genome size of *S. miscanthi* was estimated by *k*-mer analysis (*k*=19) based on Illumina DNA sequencing technology [11,12]. A short-insert library (270 bp) was constructed, and a total of ~42 Gb of clean reads was obtained for de novo assembly to estimate the whole-genome size using the standard protocol provided by the Illumina HiSeq X Ten platform. All clean reads were subjected to 19-mer frequency distribution analysis. The peak of 19-mer peak was at a depth of 89, and the genome size of *S. miscanthi* was calculated to be 393.1 Mb (Figure 2, Table 1).

Genome assembly using PacBio long reads

The genomic DNA libraries were constructed and sequenced using the PacBio Sequel platform. Additionally, 4.35 million subreads (33.88 Gb in total) with an N50 read length of 12,697 bp were obtained after removing the adaptor (Figure S1).

De novo genome assembly with long reads was performed using two pipelines, Canu and wtdbg. Because of the high heterozygosity of *Sitobion miscanthi*, in the correction step, Canu first selects longer seed reads with the settings ‘genomeSize=400000000’ and ‘corOutCoverage=50’, then detects overlapping raw reads through the highly sensitive overlapper MHAP (mhap-2.1.2, option ‘corMhapSensitivity=low/normal/high’), and finally performs an error correction with the falcon_sense method (option ‘correctedErrorRate=0.025’). In the next step, with the default parameters, error-corrected

reads are trimmed to remove unsupported bases and hairpin adapters to obtain the longest supported range. In the last step, Canu generates the draft assembly using the longest 80 coverage-trimmed reads with Canu v1.5 [13] to output more corrected reads and be more conservative at picking the error rate for the assembly to try to maintain haplotype separation. Wtdbg is an SMS data assembler that constructs fuzzy Brujin graph (available at https://github.com/ruanjue/wtdbg). Wtdbg first generates a draft assembly with the command 'wtdbg -i pbreads.fasta -t 64 -H -k 21 -S 1.02 -e 3 -o wtdbg'. The use of error-corrected reads from Canu results in better assembly performance. Then, a consensus assembly is obtained with the command 'wtdbg-cns -t 64 -i wtdbg.ctg.lay -o wtdbg.ctg.lay.fa -k 15'.

To improve genome contiguity, two assemblies generated from the Canu and wtdbg pipelines were merged with three rounds of quickmerge [14]. Quickmerge uses contigs from wtdbg as query input and contigs from Canu as ref input. The two contigs are aligned through mummer (v4.0.0, available at https://github.com/mummer4/mummer) with the nucmer parameters '-b 500 -c 100 -l 200 -t 12' and delta-filter parameters '-i 90 -r -q', and then merged through quickmerge with the parameters '-hco 5.0 -c 1.5 -l 100000 -ml 5000'. The result was error corrected using Pilon [15]. After all of the processing described above, the resulting genome assembly was further cleaned using Illumina NGS data, which were used in the 19-mer analysis above. The final draft genome assembly was 397.90 Mb, which reached a high level of continuity with a contig N50 length of 2.05 Mb (Table 2). The contig N50 of *S. miscanthi* was much higher than that of previous aphid genome assemblies constructed using DNA NGS sequencing technologies.

Genome quality evaluation

To assess the completeness of the assembled *S. miscanthi* genome, we subjected the assembled sequences to Benchmarking Universal Single-Copy Orthologs (BUSCO) version 2 [16]. Overall, 1496 and 19 of the 1658 expected Insecta genes (insect_odb9) were identified

in the assembled genome as having complete and partial BUSCO profiles, respectively. Approximately 143 genes were considered missing in our assembly. Among the expected complete Insecta genes, 1401 and 95 were identified as single-copy and duplicated BUSCOs, respectively (Figure S4).

Hi-C library construction and chromosome assembly

In this work, we used Hi-C to further assemble the genome of *S. miscanthi* at the chromosome level. Genomic DNA was extracted for the Hi-C library from the whole aphids of *S. miscanthi* mentioned above. Samples were extracted and sequenced following a standard procedure. Hi-C fragment libraries were constructed with insert sizes of 300-700bp and sequenced on the Illumina platform. Adapter sequences of raw reads were trimmed, and low-quality PE reads were removed for clean data. The clean Hi-C reads were first truncated at the putative Hi-C junctions, and then the resulting trimmed reads were aligned to the assembly results with BWA software [17]. Only uniquely alignable pairs reads whose mapping quality was more than 20 remained for further analysis. Invalid read pairs, including Dangling-End and Self-cycle, Re-ligation and Dumped products, were filtered by HiC-Pro(v2.8.1) [18].

In total, 38.44% of unique mapped read pairs were valid interaction pairs for scaffold correction and were used to cluster, order and orient scaffolds onto chromosomes by LACHESIS.

Before chromosome assembly, we first performed a preassembly for the error correction of scaffolds, which required the splitting of scaffolds into segments of 50 kb on average. The Hi-C data were mapped to these segments using BWA (version 0.7.10-r789) software. The uniquely mapped data were retained to perform assembly by using LACHESIS software. Any two segments that showed inconsistent connection with information from the raw scaffold were checked manually. These corrected scaffolds were then assembled with LACHESIS.

Parameters for running LACHESIS included CLUSTER_MIN_RE_SITES, 70; CLUSTER_MAX_LINK_DENSITY, 1; ORDER_MIN_N_RES_IN_TRUN, 19; ORDER_MIN_N_RES_IN_SHREDS, 19. After this step, placement and orientation errors exhibiting obvious discrete chromatin interaction patterns were manually adjusted. Finally, 774 scaffolds (representing 97.48% of the total length) were anchored to 9 chromosomes (Figure 3, Table S1). A genome with a final size of 377.19 Mb and a scaffold N50 of 36.26 Mb was assembled, which showed a high level of continuity with a contig N50 of 2.05 Mb using 1,167 contigs. The contig N50 of the genome assembled using PacBio long reads and Hi-C assembly was much higher than that of the 7 previously published aphid genome assemblies constructed using DNA NGS technologies (Table 3).

Repeat sequences within the S. miscanthi genome assembly

To identify tandem repeats, we utilized 4 software, namely LTR_FINDER (v1.0.5) [19], MITE-Hunter (v1.0.0)[20], RepeatScout (v1.0.5) [21], and PILER-DF (v1.0) [22] to build a de novo repeat library based on our assembly with the default settings. Subsequently, the predicted repeats were classified using PASTEClassifier (v1.0) [23] and merged with Repbase (19.06) [24]. Finally, using the resulting repeat database as the final repeat library, RepeatMasker v4.0.5 (RepeatMasker, RRID: SCR 012954) [25] was used to identify repetitive sequences in the *A. nanus* genome with the following parameters: "-nolow -no is -norna -engine wublast." The repeat sequences accounted for 31.15% of the *S. miscanthi* genome, including identified repeat sequences (26.42% of the genome), based on the de novo repeat library (Table 4).

Transcriptome sequencing to aid in gene prediction

Transcriptome sequencing (Illumina RNA-Seq and PacBio Iso-Seq) of cDNA libraries prepared from the whole newborn nymphs of *S. miscanthi* was conducted to aid in gene prediction. High-quality RNA was extracted using an SV Total RNA isolation kit (Promega,

Madison, WI, USA). Reverse transcription was completed using a Clontech SMARTer cDNA synthesis kit (Clontech Laboratories, Palo Alto, CA, USA). A paired-end library was then prepared following the Paired-End Sample Preparation Kit manual (Illumina Inc., San Diego, CA, USA). Finally, a library with an insert length of 300 bp was sequenced by an Illumina HiSeq X Ten in 150PE mode (Illumina Inc., San Diego, CA, USA). As a result, we obtained ~8.707 Gb of transcriptome data from RNA-seq. The quality of the transcripts was assessed by the proportion of gene regions covered by these transcripts, the higher being better. In this case, the proportion was 85.66%. The assembled transcripts were used to improve predictions of protein-coding genes in the *S. miscanthi* genome.

Gene annotation

Gene prediction of the *S. miscanthi* genome was performed using de novo, homology-based and transcriptome sequencing-based predictions. For de novo prediction, we employed Augustus v2.4 [26], GlimmerHMM v3.0.4 [27], SNAP (version 2006-07-28) [28], GeneID v1.4 [29] and GENSCAN [30] software to predict protein-coding genes in the *S. miscanthi* genome assembly. For homology-based prediction, protein sequences of closely related aphid species, namely, *Sipha flava*, *D. noxia*, *Ac. pisum* and *M. persicae,* were aligned against the *S. miscanthi* genome to predict potential gene structures using GeMoMa v1.3.1 [31]. For transcriptome sequencing-based prediction, we assembled the NGS transcriptome short reads into unigenes without a reference genome and then predicted genes based on unigenes using PASA v2.0.2 [32]. All of the above gene models were then integrated using EVM v1.1.1 [33] to obtain a consensus gene set. The final total gene set for the *S. miscanthi* genome was composed of 16,006 genes with an average of 6.74 exons per gene. The gene number, gene length distribution, and exon length distribution were all comparable to those of other aphid species (Table 2). Moreover, the indexes such as contig count and scaffold count were much improved.

To obtain further functional annotation of the protein-coding genes in the *S. miscanthi* genome, we employed the BLAST v2.2.31 [34] program to align the predicted genes with functional databases such as the nonredundant protein (NR) [35], EuKaryotic Orthologous Groups (KOG) [36], Gene Ontology (GO) [37], Kyoto Encyclopedia of Genes and Genomes (KEGG) [38], and Translation of European Molecular Biology Laboratory (TrEMBL) [39] databases (e-value $\leq 1e^{-5}$) (Figures S2 and S3). Ultimately, 99.35% (15,902 genes) of the 16,006 genes were annotated based on at least one database (Table S2).

Gene family identification and phylogenetic tree construction

We employed the OrthoMCL program [40] with an e-value threshold of 1e-5 to identify gene families based on the protein alignments of each gene from *S. miscanthi* and those of other insect species, which included *R. padi*, *D. noxia*, *Ac. pisum*, *M. persicae*, *Ap. glycines*, *M. cerasi*, *Rhopalosiphum maidis*, *Ap. gossypii*, *S. flava* (ftp://ftp.ncbi.nlm.nih.gov/genomes/all/GCF/003/268/045/GCF_003268045.1_YSA_version1/GCF_003268045.1_YSA_version1_genomic.fna.gz), *Apis mellifera* (ftp://ftp.ncbi.nlm.nih.gov/genomes/all/GCF/003/254/395/GCF_003254395.2_Amel_HAv3.1/GCF_003254395.2_Amel_HAv3.1_genomic.fna.gz), *D. pulex* (ftp://ftp.ncbi.nlm.nih.gov/genomes/all/GCA/000/187/875/GCA_000187875.1_V1.0/GCA_000187875.1_V1.0_genomic.fna.gz), *Drosophila melanogaster* (ftp://ftp.ncbi.nlm.nih.gov/genomes/all/GCF/000/001/215/GCF_000001215.4_Release_6_plus_ISO1_MT/GCF_000001215.4_Release_6_plus_ISO1_MT_genomic.fna.gz) and *Tribolium castaneum* (ftp://ftp.ncbi.nlm.nih.gov/genomes/all/GCF/000/002/335/GCF_000002335.3_Tcas5.2/GCF_000002335.3_Tcas5.2_genomic.fna.gz). A total of 14,722 genes were identified by clustering the homologous gene sequences from 10,918 gene families (Figure S4). One hundred thirty-eight gene families were specific to *S. miscanthi*. Subsequently, we selected 2,605 single-copy orthogroups from the abovementioned species to reconstruct the phylogenetic relationships

between *S. miscanthi* and other arthropod species. A phylogenetic tree was constructed with the maximum-likelihood method implemented in the PhyML package [41]. We used the MCMCTree program to estimate divergence times among species based on the approximate likelihood method [42] and with molecular clock data for the divergence time of medaka from the TimeTree database [43]. According to the phylogenetic analysis, *S. miscanthi* clustered with *Ac. pisum*. The divergence time between *S. miscanthi* and its common ancestor shared with *Ac. pisum* was approximately 76.8-88.4 million years (Figure 4).

Conclusions

1. We successfully assembled the chromosome-level genome of *S. miscanthi* based on long reads from the third-generation PacBio Sequel sequencing platform.

The size of the final draft genome assembly was approximately 397.91 Mb, which was slightly larger than the estimated genome size (393.12 Mb) based on *k*-mer analysis. The contigs were scaffolded onto chromosomes using Hi-C data with a contig N50 of 2.05 Mb and a scaffold N50 of 36.26 Mb. We also predicted 16,006 protein-coding genes from the generated assembly, and 99.35 (15,902 genes) of all protein-coding genes were annotated.

2. We found that the divergence time between *S. miscanthi* and its common ancestor shared with *Ac. pisum* was approximately 76.8-88.4 million years.

The assembly of this genome promotes research on the lifestyle and feeding specificity of aphids as well as their interactions with each other and other trophic levels and can serve as a resource for accelerating genome-assisted improvements in chemical drug resistant management as well as environmentally friendly aphid management.

Data availability

Data supporting the results of this article have been deposited at DDBJ/ENA/GenBank under the accession SSSL00000000. The version described in this paper is version SSSL01000000.

Declarations

List of abbreviations

BUSCO: Benchmarking Universal Single-Copy Orthologs; CDS: Coding sequence; CLR: Continuous long reads; GO: Gene Ontology; KOG: EuKaryotic Orthologous Groups; KEGG: Kyoto Encyclopedia of Genes and Genomes; LINE: Long interspersed nuclear element; LTR: Long terminal repeat; NGS: Next-generation sequencing; NR: Nonredundant protein; NT: Nonredundant nucleotide; TrEMBL: Translation of European Molecular Biology Laboratory.

Author contributions

JF and JLC conceived the project; XJ and QZ raised the aphids; XJ and YGQ collected the samples for both genome and transcriptome sequencing; QZ, XJ and JF isolated the genomic DNA for both the 19-mer analysis and genome sequencing; JF, QZ and SYZ isolated the total RNA for transcriptome sequencing; JF and HY performed the genome as well as transcriptome assembly, annotated the genome and conducted other data analysis; QL and YZ took the photographs of *S. miscanthi*; and JF and HY wrote the manuscript.

Ethics Statement

This statement is not required for experiments with *S. miscanthi*.

Acknowledgements

We thank Mr. Song Li and Huaigen Xin from Biomarker Technologies for the bioinformatics training.

Competing interests

The authors declare that they have no competing interests.

Funding

This research was sponsored by the National Key R & D Plan of China (nos. 2017YFD0200900, 2016YFD0300700 and 2017YFD0201700), the National Natural Science Foundation of China (nos. 31871966 and 31371946), the State Modern Agricultural Industry Technology System (CARS-22-G-18), and the China Scholarship Council (201703250048).

References

1. Zhang G. Aphids in agriculture and forestry of northwest China. 1st ed. Beijing: China Environmental Science. 1999.
2. The International Aphid Genomics Consortium. Genome sequence of the pea aphid *Acyrthosiphon pisum*. PLoS Biol. 2010;8:e1000313.
3. Mathers TC, Chen Y, Kaithakottil G, Legeai F, Mugford ST, Baa-Puyoulet P, et al. Rapid transcriptional plasticity of duplicated gene clusters enables a clonally reproducing aphid to colonise diverse plant species. Genome Biol. 2017;18:27.
4. Wenger JA, Cassone BJ, Legeai F, Johnston JS, Bansal R, Yates AD, et al. Whole genome sequence of the soybean aphid, *Aphis glycines*. Insect Biochem Mol Biol. 2017. doi: 10.1016/j.ibmb.2017.01.005.
5. Burger NFV, Botha AM. Genome of Russian wheat aphid an economically important cereal aphid. Stand Genomic Sci. 2017;12:90. Published 2017 Dec 28. doi:10.1186/s40793-017-0307-6
6. Thorpe P, Escudero-Martinez CM, Cock PJA, Eves-van den Akker S, Bos JIB. Shared transcriptional control and disparate gain and loss of *Aphid parasitism* genes. Genome Biol Evol. 2018;10:2716-33.
7. Quan Q, Hu X, Pan B, Zeng B, Wu N, Fang G, et al. Draft genome of the cotton *Aphid Aphis gossypii*. Insect Biochem Mol Biol. 2019;105:25-32.
8. Chen W, Shakir S, Bigham M, Fei Z, Jander G. Genome sequence of the corn leaf aphid (*Rhopalosiphum maidis* Fitch). GigaScience. 2019;8:1-12.
9. Kuznesova VG,Shaposhnikoy GKH. The chromosome numbers of the aphids (Homoptera, Aphidinea) of the world fauna. Entomological Review. 1973;52:78-96.
10. Chen X, Zhang G. The chromosome numbers of the aphids in Beijing region. Acta Zoologica Sinica. 1985;31(1):12-9.

11. Altschul SF, Gish W, Miller W, Myers EW, Lipman DJ. Basic local alignment search tool. Journal of Molecular Biology. 1990;215:403-10.

12. Li R, Li Y, Kristiansen K, Wang J. SOAP: short oligonucleotide alignment program. Bioinformatics. 2008;24:713-4.

13. Koren S, Walenz BP, Berlin K, Miller JR, Bergman NH, Phillippy AM. Canu: scalable and accurate long-read assembly via adaptive k-mer weighting and repeat separation. Genome Res. 2017;27:722-36.

14. Chakraborty M, Baldwin-Brown JG, Long AD, Emerson JJ. Contiguous and accurate de novo assembly of metazoan genomes with modest long read coverage. Nucleic Acids Res. 2016;44:e147.

15. Chin CS, Peluso P, Sedlazeck FJ, Nattestad M, Concepcion GT, Clum A, et al. Phased diploid genome assembly with single-molecule real-time sequencing. Nat Methods. 2016;13:1050-4.

16. Simão FA, Waterhouse RM, Ioannidis P, Kriventseva EV, Zdobnov EM. BUSCO: assessing genome assembly and annotation completeness with single-copy orthologs. Bioinformatics. 2015;31(19):3210-2.

17. Li H, Richard D. Fast and accurate short read alignment with Burrows–Wheeler transform. Bioinformatics. 2009;25(14):1754-60.

18. Servant, Nicolas, et al. HiC-Pro: an optimized and flexible pipeline for Hi-C data processing. Genome Biology, 2015.16(1):1-11.

19. Xu Z, Wang H. LTR_FINDER: an efficient tool for the prediction of full-length LTR retrotransposons. Nucleic Acids Res. 2007;35:W265-8.

20. Han Y, Wessler SR. MITE-Hunter: a program for discovering miniature inverted-repeat transposable elements from genomic sequences. Nucleic Acids Res. 2010;38:e199.

21. Price AL, Jones NC, Pevzner PA. De novo identification of repeat families in large

genomes. Bioinformatics. 2005;21:i351-8.
22. Edgar RC, Myers EW. PILER: identification and classification of genomic repeats. Bioinformatics. 2005;21:i152-8.
23. Hoede C, Arnoux S, Moisset M, et al. PASTEC: an automatic transposable element classification tool. PLoS One 2014;9:e91929.
24. Bao W, Kojima KK, Kohany O. Repbase Update, a database of repetitive elements in eukaryotic genomes. Mobile DNA 2015;6:11.
25. Tarailo-Graovac M, Chen N. Using RepeatMasker to identify repetitive elements in genomic sequences. Curr Protoc Bioinformatics. 2009;Chapter 4:Unit 4.10.
26. Stanke M, Waack S. Gene prediction with a hidden Markov model and a new intron submodel. Bioinformatics. 2003;19:ii215-25.
27. Majoros WH, Pertea M, Salzberg SL. TigrScan and GlimmerHMM: two open source ab initio eukaryotic gene-finders. Bioinformatics. 2004;20:2878-9.
28. Korf I. Gene finding in novel genomes. BMC Bioinformatics. 2004;5:59.
29. Blanco E, Parra G, Guigo R. Using geneid to identify genes. Curr Protoc Bioinformatics. 2007;Chapter 4:Unit 4.3.
30. Burge C, Karlin S. Prediction of complete gene structures in human genomic DNA. J Mol Biol. 1997;268:78-94.
31. Keilwagen J, Wenk M, Erickson JL, Schattat MH, Grau J, Hartung F. Using intron position conservation for homology-based gene prediction. Nucleic Acids Res. 2016;44:e89.
32. Campbell MA, Haas BJ, Hamilton JP, Mount SM, Buell CR. Comprehensive analysis of alternative splicing in rice and comparative analyses with *Arabidopsis*. BMC Genomics. 2006;7:327.
33. Haas BJ, Salzberg SL, Zhu W, Pertea M, Allen JE, Orvis J, et al. Automated eukaryotic gene structure annotation using EVidenceModeler and the program to assemble spliced

alignments. Genome Biol. 2008;9:R7.
34. Altschul SF, Gish W, Miller W, Myers EW, Lipman DJ. Basic local alignment search tool. J Mol Biol. 1990;215:403-10.
35. Marchler-Bauer A, Lu S, Anderson JB, Chitsaz F, Derbyshire MK, DeWeese-Scott C, et al. CDD: a conserved domain database for the functional annotation of proteins. Nucleic Acids Res. 2011;39:D225-9.
36. Koonin EV, Fedorova ND, Jackson JD, Jacobs AR, Krylov DM, Makarova KS, et al. A comprehensive evolutionary classification of proteins encoded in complete eukaryotic genomes. Genome Biol. 2004;5:R7.
37. Dimmer EC, Huntley RP, Alam-Faruque Y, Sawford T, O'Donovan C, Martin MJ, et al. The UniProt-GO annotation database in 2011. Nucleic Acids Res. 2012;40:D565-70.
38. Kanehisa M, Goto S. KEGG: Kyoto encyclopedia of genes and genomes. Nucleic Acids Res. 2000;28:27-30.
39. Boeckmann B, Bairoch A, Apweiler R, Blatter M-C, Estreicher A, Gasteiger E, et al. Phan I: the SWISS-PROT protein knowledgebase and its supplement TrEMBL in 2003. Nucleic Acids Res. 2003;31:365-70.
40. Li L, Stoeckert CJ, Roos DS. OrthoMCL: identification of ortholog groups for eukaryotic genomes. Genome Res. 2003;13:2178-89.
41. Guindon S, Dufayard JF, Lefort V, Anisimova M, Hordijk W, Gascuel O. New algorithms and methods to estimate maximum-likelihood phylogenies: assessing the performance of PhyML 3.0. Syst Biol. 2010;59:307-21.
42. Yang Z, Rannala B. Bayesian estimation of species divergence times under a molecular clock using multiple fossil calibrations with soft bounds. Mol Biol Evol. 2006;23:212-26.
43. Hedges SB, Marin J, Suleski M, Paymer M, Kumar S. Tree of life reveals clock-like speciation and diversification. Mol Biol Evol. 2015;32:835-45.

Figure legends

Figure 1. Winged and wingless *S. miscanthi*. a. Winged adult. b. Wingless adult.

Figure 2. 19-mer distribution for the genome size prediction of *S. miscanthi*.

Figure 3. Hi-C contact heatmap of the *S. miscanthi* genome.

Figure 4. The phylogenetic relationships of *S. miscanthi* with other arthropods.

Tables

Table 1. Assessment results based on two strategies.

Genome feature/assessment strategy	19-mer analysis	PacBio
Genome size (Mb)	393.12	397.90
GC content (%)	31.70	30.25
Repeat sequence content (%)	35.07	24.14
Heterozygosity (%)	0.98	0.57

Table 2. Assembly statistics of the *S. miscanthi* genome and 7 other aphid genomes based mainly on NGS.

Genome assembly/species	*S. miscanthi*	*R. padi*	*D. noxia*	*Ac. pisum*	*Ap. glycines*	*M. persicae*	*M. cerasi*	*Ap. gossypii*
Assembly size (Mb)	397.9	319.4	393.0	541.6	302.9	347.3	405.7	294.0
Contig count	1,148	16,689	49,357	60,623	66,000	8,249	56,508	22,569
Contig N50 (bp)	1,638,329	96,831	12,578	28,192	15,844	71,400	17,908	45,572
Scaffold count	656	15,587	5,641	23,924	8,397	4,018	49,286	4,724
Scaffold N50 (bp)	36,263,045	116,185	397,774	518,546	174,505	435,781	23,273	437,960
Genome annotation								
Gene count	16,006	26,286	19,097	36,195	17,558	18,529	28,688	14,694
Mean gene length (kb)	7.805	1,543	1.316	1.964	1.520	1.839	1,222	1.964
Mean exon count per gene	6.7	5.20	3.0	5.0	6.2	6.1	3.7	10.1
Mean exon length (bp)	288	162	249.0	394.7/429	246	299	178	218

Table 3. Summary of *S. miscanthi* genome assembly.

Statistics	Draft scaffolds	Corrected by HI-C
Contig number	1,039	1,167
Contig length	397,907,165	397,907,165
Contig N50 (bp)	2,049,770	1,565,814
Contig N90 (bp)	256,083	185,510
Contig max (bp)	11,219,273	10,100,000

Gap number/gap total length (bp)	0	0

Table 4. Detailed classification of repeats in the *S. miscanthi* genome assembly.

Type	Number	Length (bp)	Rate (%)
Class I	194093	51169345	12.86
DIRS	1,289	695,762	0.17
LINE	40,230	10,832,765	2.72
LTR/Copia	2,438	742,051	0.19
LTR/Gypsy	18,807	6,949,790	1.75
LTR/Unknown	7,534	3,195,404	0.8
PLE|LARD	115,765	28,920,417	7.27
SINE	6,665	1,075,456	0.27
SINE|TRIM	15	5,478	0
TRIM	1,116	1,281,655	0.32
Class I Unknown	234	26,384	0.01
Class II	188,820	44,184,063	11.1
Crypton	299	20,282	0.01
Helitron	5,688	1,871,785	0.47
MITE	7,972	1,434,924	0.36
Maverick	7,888	3,289,168	0.83
TIR	89,268	22,913,523	5.76
Class II Unknown	77,705	15,793,696	3.97
Potential Host Gene	926	251,812	0.06
SSR	2,611	381,142	0.1
Unknown	74,204	18,832,522	4.73
Identified	386,450	105,110,753	26.42
Total	460,654	123,943,275	31.15

Figure1

Click here to access/download;Figure;Figure 1.tiff

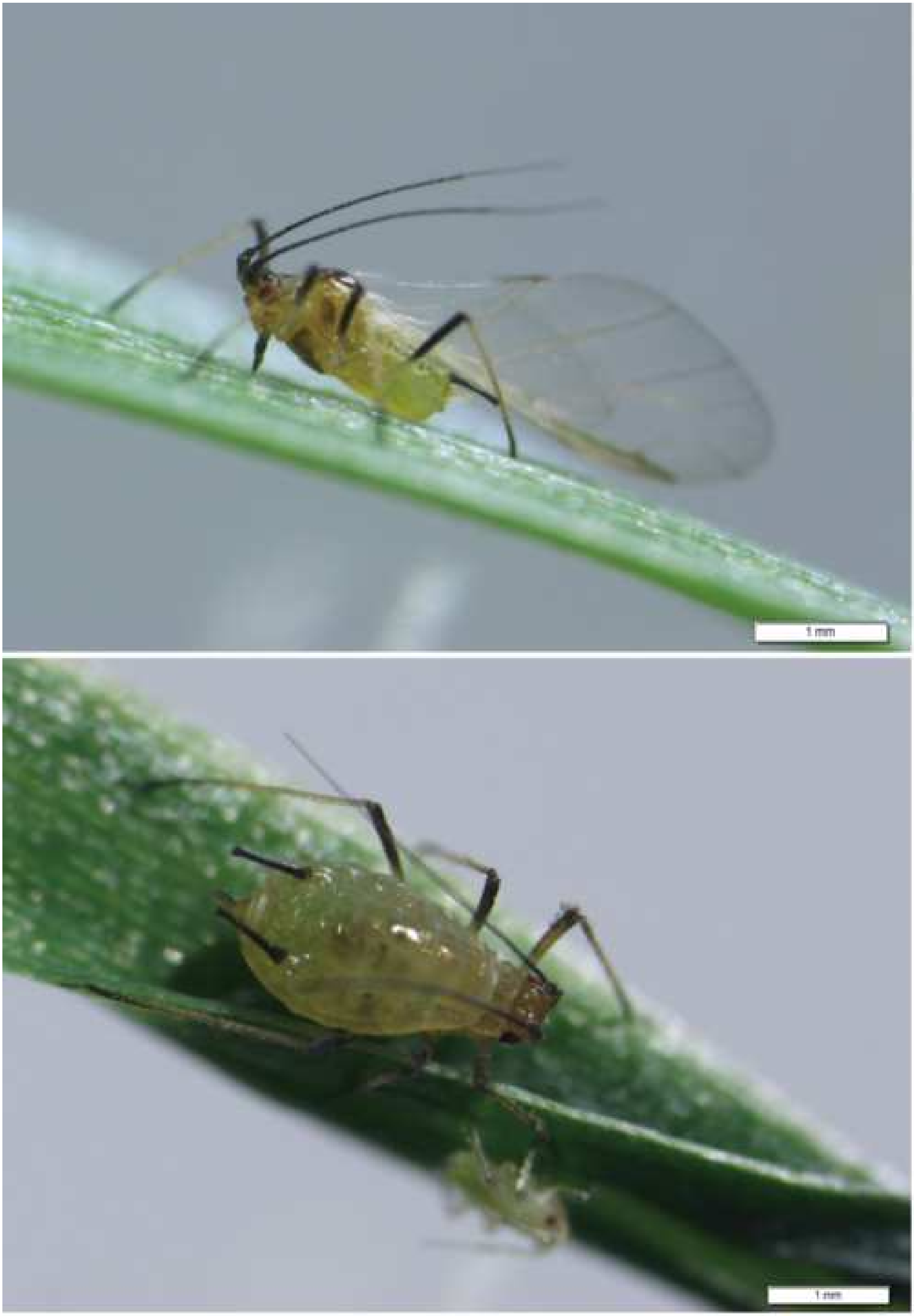

Figure2

Click here to access/download;Figure;Figure3_kmeranalysis.png

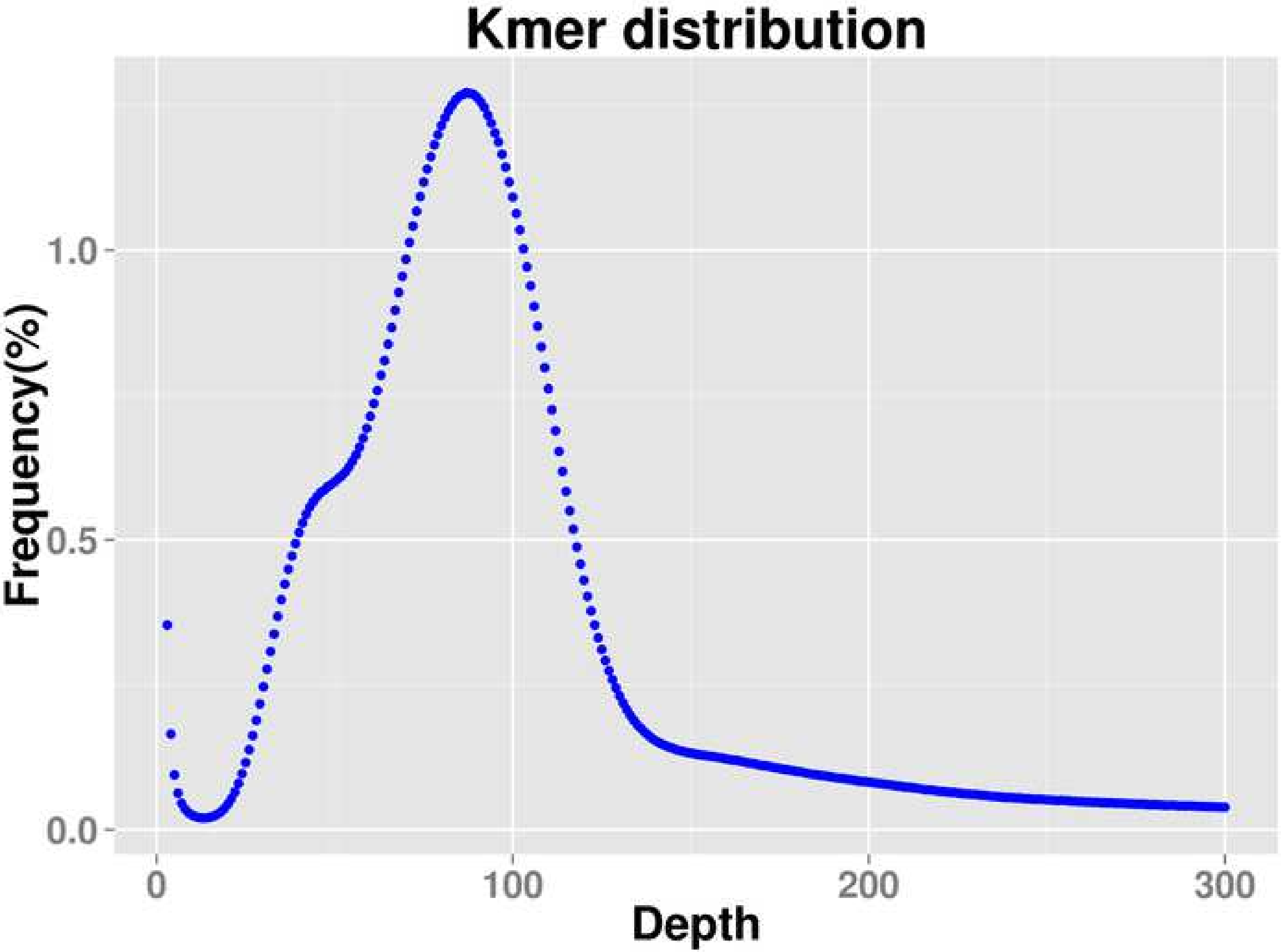

Figure3

Click here to access/download;Figure;Figure 4 HICheatmap.jpeg

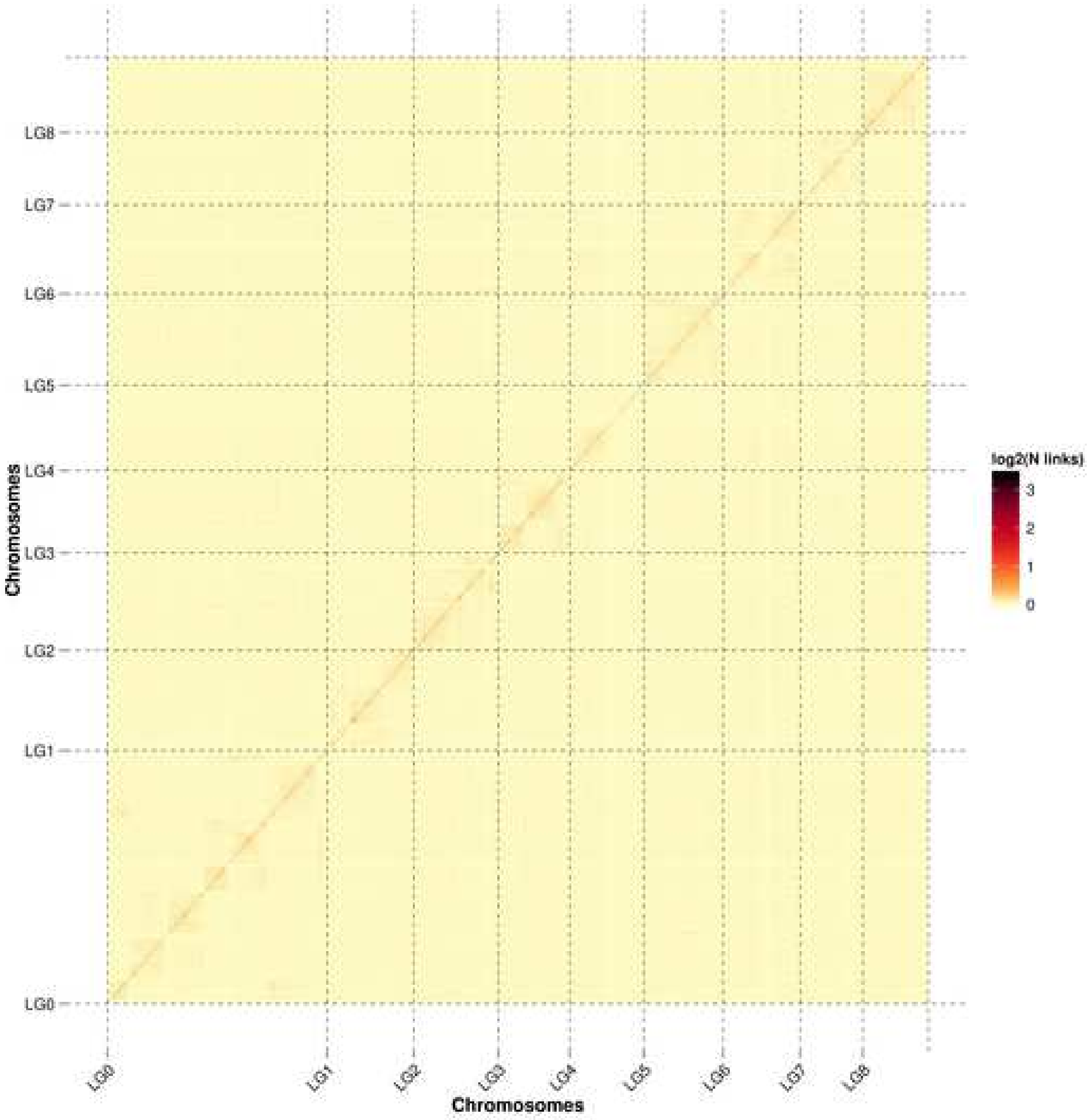

Figure4

Click here to access/download;Figure;Figure4_phylogenetic_tree_update.png

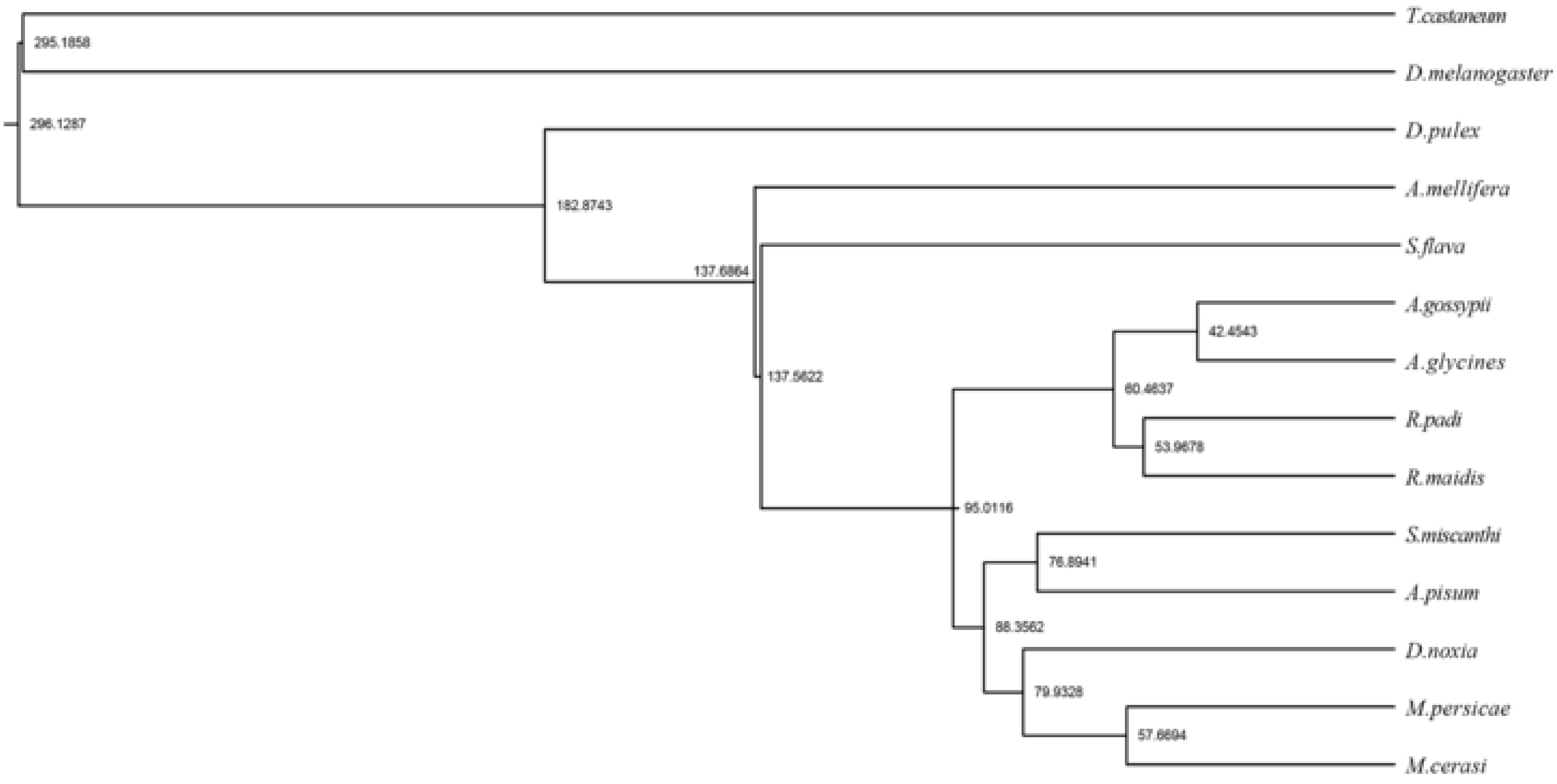

Click here to access/download
Supplementary Material
Supplementary materials0624.docx
