# Supplementary material for: A chromosome-level draft genome of the grain aphid Sitobion miscanthi
Source: Gigascience. 2019 Aug 20;8(8):giz101. doi: 10.1093/gigascience/giz101 (PMC6701489; doi:10.1093/gigascience/giz101)
Supplement: giz101_Supplemental_File [file giz101_supplemental_file.docx]

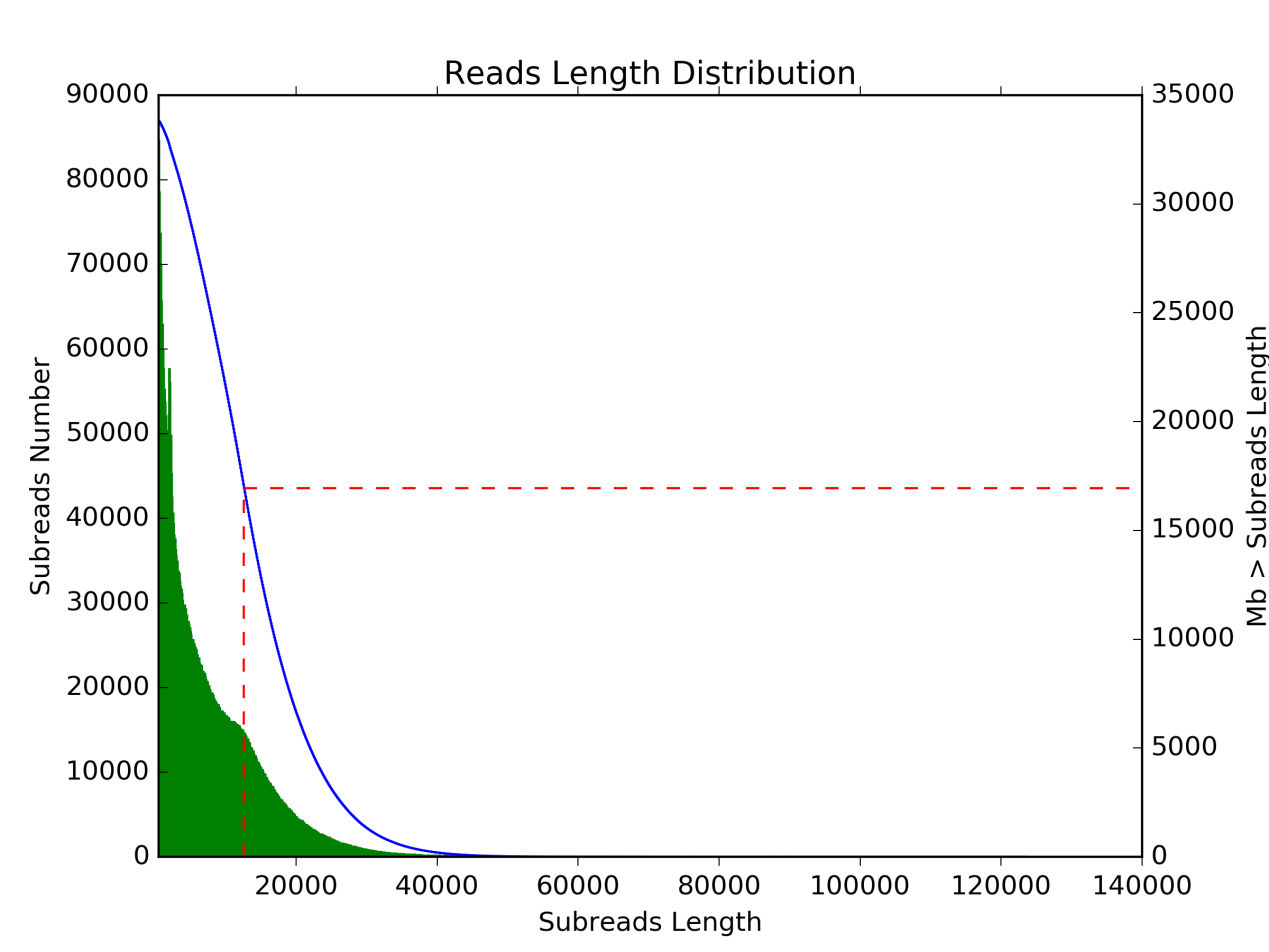


Figure S1 Filtered subread length distribution


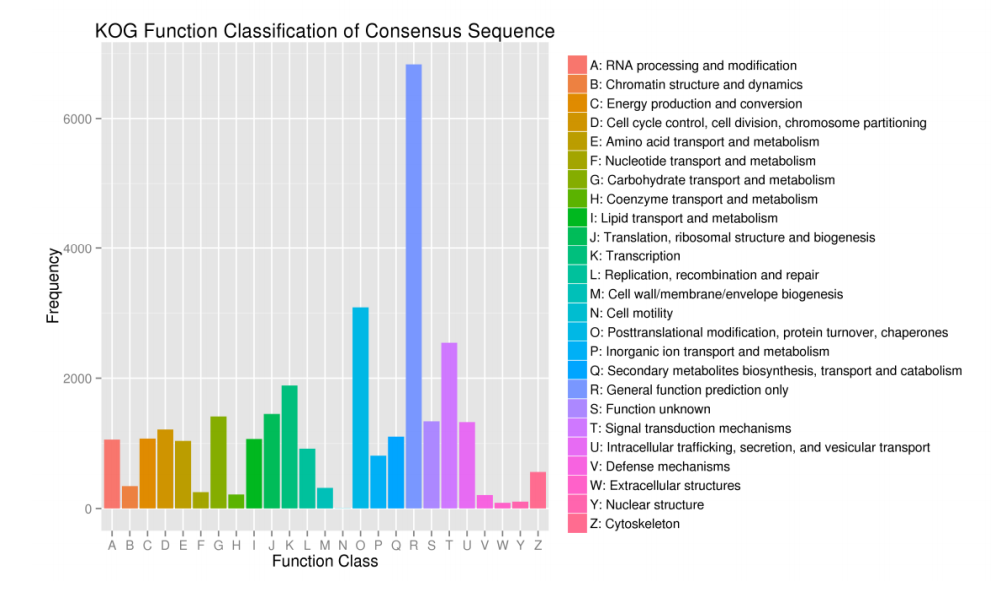


Figure S2 KOG annotation result

**
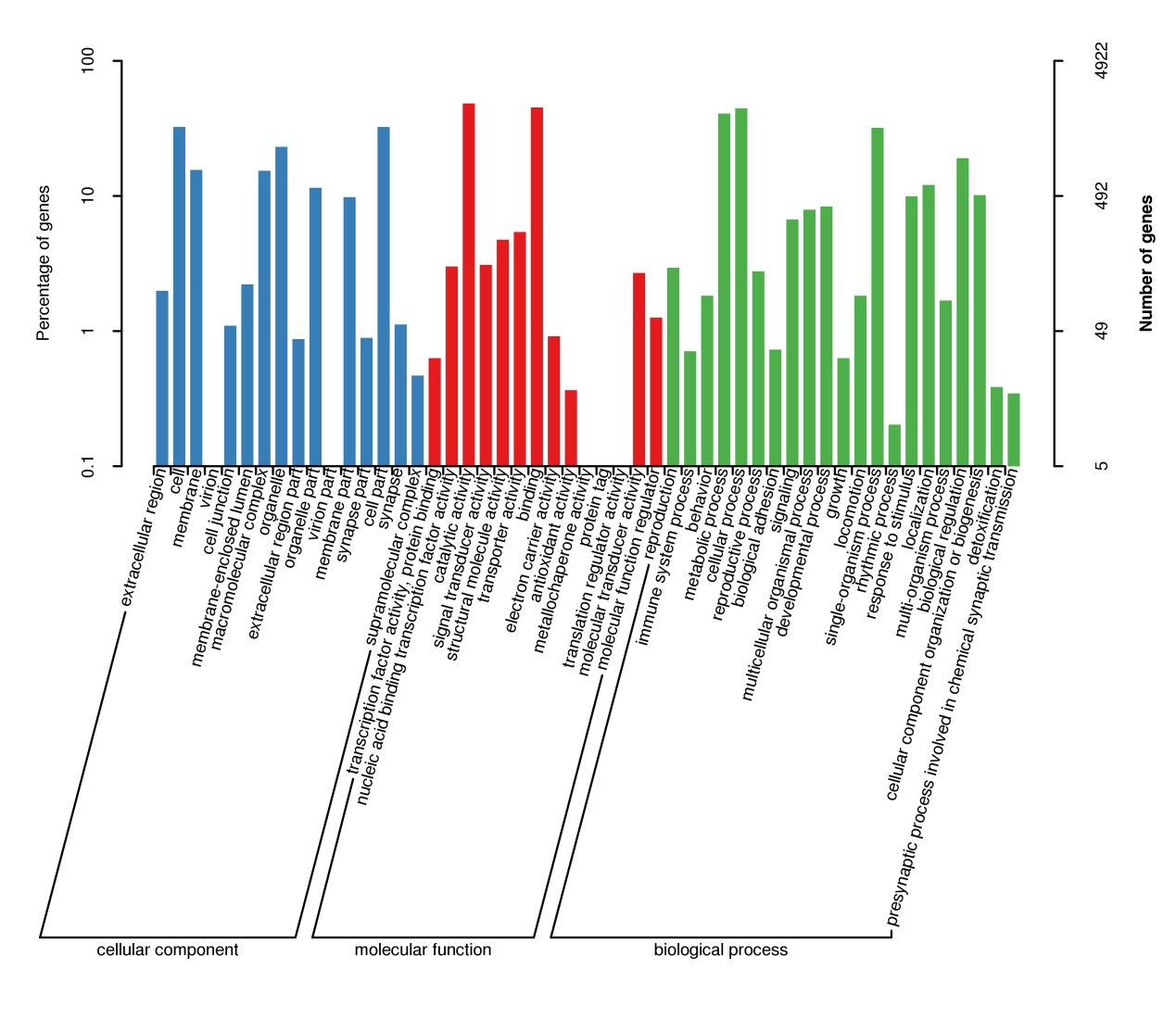
**

Figure S3 KEGG annotation result

Figure S4 Statistics of gene family clusters

Table S1 Summary of genome constructed to chromosome level of *S.avenae*

| Group | Sequence Number | Sequence Length (bp) |
| --- | --- | --- |
| Lachesis Group0 | 152 | 102338138 |
| Lachesis Group1 | 79 | 41151279 |
| Lachesis Group2 | 68 | 39866443 |
| Lachesis Group3 | 57 | 33593668 |
| Lachesis Group4 | 148 | 36747454 |
| Lachesis Group5 | 58 | 36785619 |
| Lachesis Group6 | 70 | 36121151 |
| Lachesis Group7 | 79 | 30575005 |
| Lachesis Group8 | 63 | 30712089 |
| Total Sequences Clustered (Ratio %) | 774 (67.48) | 387890846 (97.48) |
| Total Sequences Ordered and Oriented (Ratio %) | 501 (64.73) | 377194755 (97.24) |

Table S2 genome annotation

| Annotation Database | Annotated_Number | Percentage |
| --- | --- | --- |
| GO | 4922 | 30.75 |
| KEGG | 5970 | 37.30 |
| KOG | 9292 | 58.05 |
| TrEMBL | 15786 | 98.63 |
| nr | 15405 | 96.25 |
| All_Annotated | 15902 | 99.35 |
