# Supplementary material for: A chromosome-level draft genome of the grain aphid Sitobion miscanthi
Source: Gigascience. 2019 Aug 20;8(8):giz101. doi: 10.1093/gigascience/giz101 (PMC6701489; doi:10.1093/gigascience/giz101)
Supplement: giz101_Response_to_Reviewer_Comments_Original_Submission [file giz101_response_to_reviewer_comments_original_submission.pdf]

Author's Response To Reviewer Comments

Close

Dear editor and reviewers,

Thank you for your concerning on our manuscript entitled “A chromosome-level draft genome of the grain aphid Sitobion miscanthi” (Manuscript ID: GIGA-D-19-00137). We appreciate very much for your helpful comments and constructive suggestions on this manuscript. Those comments are all very helpful for us to improve our manuscript. Please receive the revised manuscript. All the co-authors and I have carefully checked the manuscript and revised it according to your suggestions and the comments.
Please let us know if you have any question or need any further information about our manuscript.
Sincerely yours,

Jia Fan
Dr. Jia Fan
State Key Laboratory for Biology of Plant Diseases and Insect Pests
Institute of Plant Protection
Chinese Academy of Agricultural Sciences
Beijing 100193, China
Email: jfan@ippcaas.cn

Reviewer 1:
The authors describe the genome of S. miscanthi using modern HiSeq methods. This manuscript and these data will add to the rapidly growing number of important aphid pest species with complete genome sequences. While the data appears solid, and the genome is high quality, I have some major comments on the manuscript:
Major:
1. The manuscript seems to weave two tales, but only one (the genome) is strong enough for publication. The authors try to incorporate what appears to be a sibling species issue, with very light discussion and detail on apparent mis-identification or confusion between S. miscanthi and S. avenae. They tried to add some survey data (with collections in 17 different Chinese locations), but this section does not include any data as far as sampling, variation, etc. While the troubling taxonomy may be an issue for this system the authors need to make a decision to either remove this section and focus on the genome, or be ready to add more detail to the manuscript to support their claim of S. miscanthi. To keep the manuscript simple, I suggest that the authors follow the former—the inclusion of the population data is not necessary, in my opinion, for publication.
Response: According to the reviewer’s suggestions, we have removed the species identification section to make the manuscript simple and focus on the genome.
2. In many bioinformatic analyses, the authors seem to not have a workflow, and rather just use any sequencing software that may fit their data. While it is ok to use multiple programs, using them without a rationale makes it seem like the authors are grasping at straws. Furthermore, there are no details for each program? Why did they use all the programs? What did they expect to see and why did they not see it initially and then had to use a different program? How do they know they got an adequate answer with one program and not the other? For example, the section starting on L148 contains multiple programs without any output, rationale or discussion on the different programs.
Response:We have added more detail in the sections on “genome assembly” as well as “annotation” in our manuscript (Line 87-108):
De novo genome assembly with long reads was performed using two pipelines, Canu and wtdbg. Because of the high heterozygosity of Sitobion miscanthi, in the correction step, Canu first selects longer seed reads with the settings ‘genomeSize=400000000’ and ‘corOutCoverage=50’, then detects overlapping raw reads through the highly sensitive overlapper MHAP (mhap-2.1.2, option ‘corMhapSensitivity=low/normal/high’), and finally performs error correction with the falcon_sense method (option ‘correctedErrorRate=0.025’). In the next step, with the default parameters, error-corrected reads are trimmed to remove unsupported bases and hairpin adapters to obtain the longest supported range. In the last step, Canu generates the draft assembly using the longest 80 coverage-

trimmed reads with Canu v1.5 [13] to output more corrected reads and be more conservative at picking the error rate for the assembly to try to maintain haplotype separation.
Wtdbg is an SMS data assembler that constructs fuzzy Brujin graphs (available at https://github.com/ruanjue/wtdbg). Wtdbg first generates a draft assembly with the command 'wtdbg -i pbreads.fasta -t 64 -H -k 21 -S 1.02 -e 3 -o wtdbg'. The use of error-corrected reads from Canu results in better assembly performance. Then, a consensus assembly is obtained with the command 'wtdbg-cns -t 64 -i wtdbg.ctg.lay -o wtdbg.ctg.lay.fa -k 15'.
To improve genome contiguity, two assemblies generated from the Canu and wtdbg pipelines were merged with three rounds of quickmerge [14]. Quickmerge uses contigs from wtdbg as query input and contigs from Canu as ref input. The two contigs are aligned through mummer (v4.0.0, available at https://github.com/mummer4/mummer), with the nucmer parameters '-b 500 -c 100 -l 200 -t 12' and delta-filter parameters '-i 90 -r -q', and then merged through quickmerge with the parameters '-hco 5.0 -c 1.5 -l 100000 -ml 5000'.

Minor Comments
3. l21: is a worldwide, sap-sucking pest of wheat; l24: "This species was..." this sentence is probably not relevant to include in the abstract. Instead replace the sentence with one that explains why the genome would be so important.
Response: We revised this as follows (from Line 21 to Line 24):
Sitobion miscanthi is an ideal model for studying host plant specificity, parthenogenesis-based phenotypic plasticity, and interactions between insects and other species of various trophic levels, such as viruses, bacteria, plants and natural enemies. However, the genome information for this species has not been published yet.
4. l40: This sentence needs to be broken up, it is far too long and contains several different ideas
Response: This sentence has been broken up as follows (Line 35-39):
We generated a high-quality draft of the Sitobion miscanthi genome. This genome assembly promotes research on the lifestyle and feeding specificity of aphids and their interactions with each other and species at other trophic levels. It can serve as a resource for accelerating genome-assisted improvements in chemical drug resistant management and environmentally friendly aphid management.
5. l53: remove product
l54: replace scope with range, and replace the simple with its simple
l55: ideal model is redundant
l62: remove are available. Furthermore, I don't think you need to list where these genomes are since each aphid genome is properly cited
l63: italicize A .gossypii
Response: We thank for the reviewer's very meticulous review. According to the reviewer's comments, they were revised as follows:
In line 51 of revised manuscript, product was removed.
Scope was replaced with range in line 48, and the simple was replaced with its simple in line 48-49.
In line 50, ideal model was changed into significant.
Fully consider the reviewer's helpful advice, this sentence (Line 52-56) was re-organized: Genomes with annotation information from a total of 8 aphid species, namely the pea aphid Acyrthosiphon pisum [2], peach aphid Myzus persicae [3], soybean aphid Aphis glycines [4], Russian wheat aphid Diuraphis noxia [5], cherry-oat aphid Rhopalosiphum padi [6], and black cherry aphid Myzus cerasi [6], the cotton aphid Aphis gossypii [7], and the corn leaf aphid Rhopalosiphum maidis [8] are available.
In line 59, Aphis gossypii is italicize now.
6. L79: How do the authors know that nymphs collected within 12 h did not feed?
Response: Mother aphids were placed into culture dishes (dimeter of 9 cm) with moist absorbent paper on the bottom for 12 h. No newborn nymphs were fed during this period. Newborn nymphs within 12 h without feeding were collected for genome sequencing. We have added this sentence into the manuscript to clarify(Line 67-70). Thank you very much.
7. Figure 2: What am I looking at here? The figures have labels but the caption needs more detail to explain to the reader what is relevant
Response: According to the reviewer's first comment, the species identification section was removed from this manuscript.
8. L93: This section needs a better citation for the methods other than including a protocol
Response: According to the reviewer's comment, two citations cited as [11] and [12] have been inserted into the manuscript (Line 77).
11. Altschul SF, Gish W, Miller W, Myers EW, Lipman DJ. Basic local alignment search tool. Journal of Molecular Biology. 1990;215:403-10.
12. Li R, Li Y, Kristiansen K, Wang J. SOAP: short oligonucleotide alignment program. Bioinformatics. 2008;24:713-14.

9. L99: remove finally
Response: We removed "finally" (Line 78).
10. L129: I think more detail is needed here for Hi-C sequencing other than just citing 2 different papers
Response: According to the reviewer's suggestion, we have detailed the Hi-C sequencing in line 127-149 as follows,
Hi-C fragment libraries were constructed with insert sizes of 300-700 bp and sequenced on the Illumina platform. Adapter sequences of raw reads were trimmed, and low-quality PE reads were removed for clean data. The clean Hi-C reads were first truncated at the putative Hi-C junctions, and then the resulting trimmed reads were aligned to the assembly results with BWA software [17]. Only uniquely alignable pairs reads whose mapping quality was more than 20 remained for further analysis. Invalid read pairs, including Dangling-End and Self-cycle, Re-ligation and Dumped products, were filtered by HiC-Pro (v2.8.1) [18].
In total, 38.44% of unique mapped read pairs were valid interaction pairs for scaffold correction and were used to cluster, order and orient scaffolds onto chromosomes by LACHESIS.
Before chromosome assembly, we first performed a preassembly for the error correction of scaffolds, which required the splitting of scaffolds into segments of 50 kb on average. The Hi-C data were mapped to these segments using BWA (version 0.7.10-r789) software. The uniquely mapped data were retained to perform assembly by using LACHESIS software. Any two segments that showed inconsistent connections with information from the raw scaffold were checked manually. These corrected scaffolds were then assembled with LACHESIS. Parameters for running LACHESIS included CLUSTER_MIN_RE_SITES, 70; CLUSTER_MAX_LINK_DENSITY, 1; ORDER_MIN_N_RES_IN_TRUN, 19; ORDER_MIN_N_RES_IN_SHREDS, 19. After this step, placement and orientation errors exhibiting obvious discrete chromatin interaction patterns were manually adjusted. Finally, 774 scaffolds (representing 97.48% of the total length) were anchored to 9 chromosomes (Figure 3, Table S1).
11. L132: BWA software needs a citation
Response: According to the reviewer's comment, one citation of [17] has been inserted into the manuscript (Line 131).
17. Li H, Richard D. Fast and accurate short read alignment with Burrows–Wheeler transform. Bioinformatics. 2009;25(14):1754-60.
12. Figure 4 is unreadable.
Response: We are very sorry for this mistake. Figure 4 has been uploaded again, and it is also shown below.

13. L140: do they have a karyotype citation?
Response: Yes, two citations of [9] and [10] have been inserted into the manuscript (Line 61).
9. Kuznesova VG, Shaposhnikoy GKH. The chromosome numbers of the aphids (Homoptera, Aphidinea) of the world fauna. Entomological Review. 1973;52:78-96.
10. Chen XD, Zhang GX. The chromosome numbers of the aphids in Beijing region.Acta Zoologica Sinica. 1985;31(1):12-9.
14. L147: Similar to the assembly, why all these programs? And what data did all these programs give (and where is it)? What is the workflow for this part of the study?
Response: We have added more details for this part from line 155 to line 164.
To identify tandem repeats, we utilized 4 software programs, namely LTR_FINDER (v1.0.5) [19], MITE-Hunter (v1.0.0) [20], RepeatScout (v1.0.5) [21], and PILER-DF (v1.0) [22], to build a de novo repeat library based on our assembly with the default settings. Subsequently, the predicted repeats were classified using PASTEClassifier (v1.0) [23] and merged with Repbase (19.06) [24]. Finally, using the resulting repeat database as the final repeat library, RepeatMasker v4.0.5 (RepeatMasker, RRID: SCR 012954) [25] was used to identify repetitive sequences in the A. nanus genome with the following parameters: "-nolow -no is -norna -engine wublast." The repeat sequences accounted for 31.15% of the S. miscanthi genome, including identified repeat sequences (26.42% of the genome), based on the de novo repeat library (Table 4).
15. L165: How were the transcript assembled? And how good is this assembly?
Response: We have detailed the method of transcript assembled and quality assessed in line 174-176.
Transcripts were assembled based on the genome-guide trinity.
The quality of the transcripts was assessed by the proportion of gene regions covered by these transcripts, the higher being better. In this case, the proportion was 85.66%.
16. L235: remove the ,
Response: According to the reviewer's comments, we have removed "the" (Line 241).
17. Figure 5 is poor quality, I cannot read the node support. Plus, S. avenae is included--I assume this is miscanthi?
Response: We are very sorry for the poor quality of Figure 5, and the current Figure 5 is updated as follows:

Reviewer 2:
The authors sequenced the genome of Sitobion miscanthi using long-read sequencing and Hi-C. They generated chromosome-length scaffolds and assembled the collected data to produce nine chromosomes to a final size of 377.19 Mb. A total of 16,006 PCGs were annotated and found that 26.41% of the genome consists of repetitive sequences. The manuscript presents a good genome draft of a Hemipteran species, previously not reported and as such merit publication. However, the paper contains many discrepancies that require addressing.
1. Also the grammar should be improved.
Responses: According to the reviewer's suggestion, to improve our manuscript, the revised version was edited by a professional language editor before submission.
2. The legend of fig 2 should be improved as it is unclear which samples represent S. avenae and S. marcanthi.
Response:The species identification section, including Figure 2, was removed to simplify this manuscript and focus on the genome.
3. The phylogenetic analysis (Fig 5) provides insight into the relationships of different arthropod species relative to S. miscanthi, but S. miscanthi doesn't feature in the figure? So, I fail to see what the value of this figure is. The resolution of the figure should also be improved as it is impossible to read the values on the branches.
Response: We are very sorry for this, and the current Figure 5 was updated and uploaded in the submission system.
4. Table 1 give the statistics of all the sequenced aphid genomes, but doesn't include the most recent/updated versions of the different genomes (e.g. D. noxia actually has more genes, see the paper in Standards in Genomic Sciences 2018, https://doi.org/ 10.1186/s40793-017-0307-6). Please update to represent the most recent data.
Response: The most recent data have been updated in Table 2.
5. It is interesting that the genome size estimated on k-mer analysis differs so much from the final genome estimation, please explain/discuss.
Response: The genome size assessed by the survey map is 393.12 Mb, and the final genome size is 397.95 Mb. The overall rate of heterozygosity (~0.98) indicated that this genome may have a high rate of heterozygous regions, which should be carefully considered in subsequent assembly processes. A Canu assembly of 585.29 Mb and 6,014 contigs were generated, with most haplotypes associated with diploid populations. High heterozygosity of the genome is the main cause of a large genome assembly.
6. Sipha flava is not listed as a genome with sequence information - so where were the data obtained from to conduct the homology-based predictions? Please clarify.
Response: This species, Sipha flava, cited in our manuscript is the only aphid whose genome has not been formally published. The website where its sequence information can be downloaded is shown in our manuscript (Lines 206-207)
ftp://ftp.ncbi.nlm.nih.gov/genomes/all/GCF/003/268/045/GCF_003268045.1_YSA_version1 196 /GCF_003268045.1_YSA_version1_genomic.fna.gz)

Minor issues:
7. Lines 59-64, and again line 145: "information of 6 aphid species"' - but I count 8?

Response: According to the reviewer's comments, we have carefully corrected them all (Line 52, 152).
8. Line 188: Refers to functional annotation of PCGs but cite Fig. 3 (k-mer analysis)?
Response: Yes, it should be Figure S3, we have corrected it in the manuscript (Line 199). We invited a language editor to minimize similar editorial issues.
9. There are many minor editorial issues that needs addressing (e.g., lack of commas, lines 59-64; in appropriate comma use, line 235; not using the demonstrative pronoun "the", line 73 etc.,)
Response: We invited a language editor to minimize similar editorial issues. Thank you very much.

Close
