# Supplementary material for: A chromosome-level draft genome of the grain aphid Sitobion miscanthi
Source: Gigascience. 2019 Aug 20;8(8):giz101. doi: 10.1093/gigascience/giz101 (PMC6701489; doi:10.1093/gigascience/giz101)
Supplement: giz101_Response_to_Reviewer_Comments_Revision_1 [file giz101_response_to_reviewer_comments_revision_1.pdf]

Author's Response To Reviewer Comments

Close

July 19, 2019

Dear editor,

Thank you for your email on July 19, 2019 regarding the decision of our manuscript entitled "A chromosome-level draft genome of the grain aphid Sitobion miscanthi" (Manuscript ID: GIGA-D-19-00137R1). I appreciate very much for your suggestions on the manuscript. I also acknowledge the two reviewers for their kind comments on this manuscript.
All the co-authors and I have carefully checked the manuscript and revised it according to your suggestions and the reviewers' comments. In the revised manuscript, we have revised words (in lines 38 & 239 and lines 39 & 240) and sentence (current line 44-45) to improve them clearly presented. Please receive the revised manuscript.
Thank you for your consideration and your time.
Please let us know if you need any further information about our manuscript.
Sincerely yours,

Jia Fan
Dr. Jia Fan
State Key Laboratory for Biology of Plant Diseases and Insect Pests
Institute of Plant Protection
Chinese Academy of Agricultural Sciences
Beijing 100193, China
Email: jfan@ippcaas.cn

Reviewer 1:
1. L38 & 239: I assume the authors mean insecticide resistance management?
Response: Yes, according to the reviewer's comments, "chemical drug" resistant management has been replaced by "insecticide" resistance management (Line38 and Line 237).

2. L39 & 240: replace friendly with safe
Response: Thank for the reviewer's correction, "friendly" has been replaced by "safe" (Line39 and Line 238).

3. L50-52: These two sentences make it seem like the authors published the genome because of the misidentification. Is this the rationale for the genome?
Response: We thank for the reviewer's suggestion. According to the reviewer's comments, the sentence "This species was misidentified as Sitobion avenae in China [1]." was inserted into the first sentence as follows (Line44-45):
The grain aphid Sitobion miscanthi (Figure 1) widely mis-reported as Sitobion avenae in China [1], is a globally distributed sap-sucking specialist of cereal and a dominant species in wheat-growing regions across China.

Close
