# Supplementary material for: A chromosome-level draft genome of the grain aphid Sitobion miscanthi
Source: Gigascience. 2019 Aug 20;8(8):giz101. doi: 10.1093/gigascience/giz101 (PMC6701489; doi:10.1093/gigascience/giz101)
Supplement: giz101_Reviewer_1_Report_Original_Submission — Andrew Michel -- 5/20/2019 Reviewed [file giz101_reviewer_1_report_original_submission.pdf]

Reviewer Report

Title: A chromosome-level draft genome of the grain aphid Sitobion miscanthi

Version: Original Submission **Date:** 5/20/2019

Reviewer name: Andrew Michel

Reviewer Comments to Author:

The authors describe the genome of S. miscanthi using modern HiSeq methods. This manuscript and these data will add to the rapidly growing number of important aphid pest species with complete genome sequences. While the data appears solid, and the genome is high quality, I have some major comments on the manuscript:

Major:

The manuscript seems to weave two tales, but only one (the genome) is strong enough for publication. The authors try to incorporate what appears to be a sibling species issue, with very light discussion and detail on apparent mis-identification or confusion between S. miscanthi and S. avenae. They tried to add some survey data (with collections in 17 different Chinese locations), but this section does not include any data as far as sampling, variation, etc. While the troubling taxonomy may be an issue for this system the authors need to make a decision to either remove this section and focus on the genome, or be ready to add more detail to the manuscript to support their claim of S. miscanthi. To keep the manuscript simple, I suggest that the authors follow the formerâ€"the inclusion of the population data is not necessary, in my opinion, for publication.

In many bioinformatic analyses, the authors seem to not have a workflow, and rather just use any sequencing software that may fit their data. While it is ok to use multiple programs, using them without a rationale makes it seem like the authors are grasping at straws. Furthermore, there are no details for each program? Why did they use all the programs? What did they expect to see and why did they not see it initially and then had to use a different program? How do they know they got an adequate answer with one program and not the other? For example, the section starting on L148 contains multiple programs without any output, rationale or discussion on the different programs.

Minor Comments

l21: is a worldwide, sap-sucking pest of wheat

l24: "This species was..." this sentence is probably not relevant to include in the abstract. Instead replace the sentence with one that explains why the genome would be so important.

l40: This sentence needs to be broken up, it is far too long and contains several different ideas

l53: remove product

l54: replace scope with range, and replace the simple with its simple

l55: ideal model is redundant

l62: remove are available. Furthermore, I don't think you need to list where these genomes are since each aphid genome is properly cited

l63: italicize A .gossypii

L79: How do the authors know that nymphs collected within 12 h did not feed?

Figure 2: What am I looking at here? The figures have labels but the caption needs more detail to explain to the reader what is relevant
L93: This section needs a better citation for the methods other than including a protocol
L99: remove finally
L129: I think more detail is needed here for Hi-C sequencing other than just citing 2 different papers
L132: BWA software needs a citation
Figure 4 is unreadable.
L140: do they have a karyotype citation?
L147: Similar to the assembly, why all these programs? And what data did all these programs give (and where is it)? What is the workflow for this part of the study?
L165: How were the transcript assembled? And how good is this assembly?
L235: remove the ,
Figure 5 is poor quality, I cannot read the node support. Plus, S. avenae is included--I assume this is miscanthi?

Level of Interest

Please indicate how interesting you found the manuscript: Choose an item.

Quality of Written English

Please indicate the quality of language in the manuscript: Choose an item.

Declaration of Competing Interests

Please complete a declaration of competing interests, considering the following questions:

- Have you in the past five years received reimbursements, fees, funding, or salary from an organisation that may in any way gain or lose financially from the publication of this manuscript, either now or in the future?
- Do you hold any stocks or shares in an organisation that may in any way gain or lose financially from the publication of this manuscript, either now or in the future?
- Do you hold or are you currently applying for any patents relating to the content of the manuscript?
- Have you received reimbursements, fees, funding, or salary from an organization that holds or has applied for patents relating to the content of the manuscript?
- Do you have any other financial competing interests?
- Do you have any non-financial competing interests in relation to this paper?

If you can answer no to all of the above, write 'I declare that I have no competing interests' below. If your reply is yes to any, please give details below.

I declare that I have no competing interests

I agree to the open peer review policy of the journal. I understand that my name will be included on my report to the authors and, if the manuscript is accepted for publication, my named report including any attachments I upload will be posted on the website along with the authors' responses. I agree for my report to be made available under an Open Access Creative Commons CC-BY license (http://creativecommons.org/licenses/by/4.0/). I understand that any comments which I do not wish to be included in my named report can be included as confidential comments to the editors, which will not be published.

I agree to the open peer review policy of the journal

To further support our reviewers, we have joined with Publons, where you can gain additional credit to further highlight your hard work (see: https://publons.com/journal/530/gigascience). On publication of this paper, your review will be automatically added to Publons, you can then choose whether or not to claim your Publons credit. I understand this statement.

Yes Choose an item.
