# Supplementary material for: A chromosome-level draft genome of the grain aphid Sitobion miscanthi
Source: Gigascience. 2019 Aug 20;8(8):giz101. doi: 10.1093/gigascience/giz101 (PMC6701489; doi:10.1093/gigascience/giz101)
Supplement: giz101_Reviewer_2_Report_Original_Submission — Anna-Maria Botha -- 6/5/2019 Reviewed [file giz101_reviewer_2_report_original_submission.pdf]

Reviewer Report

Title: A chromosome-level draft genome of the grain aphid Sitobion miscanthi

Version: Original Submission **Date:** 6/5/2019

Reviewer name: Anna-Maria Botha

Reviewer Comments to Author:

The authors sequenced the genome of Sitobion miscanthi using long-read sequencing and Hi-C. They generated chromosome-length scaffolds and assembled the collected data to produce nine chromosomes to a final size of 377.19 Mb. A total of 16,006 PCGs were annotated and found that 26.41% of the genome consists of repetitive sequences. The manuscript presents a good genome draft of a Hemipteran species, previously not reported and as such merit publication. However, the paper contains many discrepancies that require addressing. Also the grammar should be improved.
The legend of fig 2 should be improved as it is unclear which samples represent S. avenae and S. marcanthi.
The phylogenetic analysis (Fig 5) provides insight into the relationships of different arthropod species relative to S. miscanthi, but S. miscanthi doesn't feature in the figure? So, I fail to see what the value of this figure is. The resolution of the figure should also be improved as it is impossible to read the values on the branches.
Table 1 give the statistics of all the sequenced aphid genomes, but doesn't include the most recent/updated versions of the different genomes (e.g. D. noxia actually has more genes, see the paper in Standards in Genomic Sciences 2018, https://doi.org/ 10.1186/s40793-017-0307-6). Please update to represent the most recent data.
It is interesting that the genome size estimated on k-mer analysis differs so much from the final genome estimation, please explain/discuss.
Sipha flava is not listed as a genome with sequence information - so where were the data obtained from to conduct the homology-based predictions? Please clarify.
Minor issues:
Lines 59-64, and again line 145: "information of 6 aphid species"' - but I count 8?
Line 188: Refers to functional annotation of PCGs but cite Fig. 3 (k-mer analysis)?
There are many minor editorial issues that needs addressing (e.g., lack of commas, lines 59-64; in appropriate comma use, line 235; not using the demonstrative pronoun "the", line 73 etc.,)

Level of Interest

Please indicate how interesting you found the manuscript: Choose an item.

Quality of Written English

Please indicate the quality of language in the manuscript: Choose an item.

Declaration of Competing Interests

Please complete a declaration of competing interests, considering the following questions:

- Have you in the past five years received reimbursements, fees, funding, or salary from an organisation that may in any way gain or lose financially from the publication of this manuscript, either now or in the future?
- Do you hold any stocks or shares in an organisation that may in any way gain or lose financially from the publication of this manuscript, either now or in the future?
- Do you hold or are you currently applying for any patents relating to the content of the manuscript?
- Have you received reimbursements, fees, funding, or salary from an organization that holds or has applied for patents relating to the content of the manuscript?
- Do you have any other financial competing interests?
- Do you have any non-financial competing interests in relation to this paper?

If you can answer no to all of the above, write 'I declare that I have no competing interests' below. If your reply is yes to any, please give details below.

Not applicable

I agree to the open peer review policy of the journal. I understand that my name will be included on my report to the authors and, if the manuscript is accepted for publication, my named report including any attachments I upload will be posted on the website along with the authors' responses. I agree for my report to be made available under an Open Access Creative Commons CC-BY license (http://creativecommons.org/licenses/by/4.0/). I understand that any comments which I do not wish to be included in my named report can be included as confidential comments to the editors, which will not be published.

I agree to the open peer review policy of the journal

To further support our reviewers, we have joined with Publons, where you can gain additional credit to further highlight your hard work (see: https://publons.com/journal/530/gigascience). On publication of this paper, your review will be automatically added to Publons, you can then choose whether or not to claim your Publons credit. I understand this statement.

Yes Choose an item.
